# Similarities and differences in the regulation of *HoxD* genes during chick and mouse limb development

**DOI:** 10.1371/journal.pbio.3000004

**Published:** 2018-11-26

**Authors:** Nayuta Yakushiji-Kaminatsui, Lucille Lopez-Delisle, Christopher Chase Bolt, Guillaume Andrey, Leonardo Beccari, Denis Duboule

**Affiliations:** 1 School of Life Sciences, Federal Institute of Technology, Lausanne, Lausanne, Switzerland; 2 Department of Genetics and Evolution, University of Geneva, Geneva 4, Switzerland; University of Zurich, SWITZERLAND

## Abstract

In all tetrapods examined thus far, the development and patterning of limbs require the activation of gene members of the *HoxD* cluster. In mammals, they are regulated by a complex bimodal process that controls first the proximal patterning and then the distal structure. During the shift from the former to the latter regulation, this bimodal regulatory mechanism allows the production of a domain with low *Hoxd* gene expression, at which both telomeric (T-DOM) and centromeric regulatory domains (C-DOM) are silent. These cells generate the future wrist and ankle articulations. We analyzed the implementation of this regulatory mechanism in chicken, i.e., in an animal for which large morphological differences exist between fore- and hindlimbs. We report that although this bimodal regulation is globally conserved between the mouse and the chick, some important modifications evolved at least between these two model systems, in particular regarding the activity of specific enhancers, the width of the TAD boundary separating the two regulations, and the comparison between the forelimb versus hindlimb regulatory controls. At least one aspect of these regulations seems to be more conserved between chick and bats than with mouse, which may relate to the extent to which forelimbs and hindlimbs of these various animals differ in their morphologies.

## Abstract 2

A comparison of Hox gene regulation during the development of limbs in birds and mammals reveals that whereas the characteristic bimodal regulatory system, based on large chromatin domains, is largely conserved between these morphologically distinct structures, some differences are revealed in the way this is implemented in various vertebrates.

## Author summary

The shapes of limbs vary greatly among tetrapod species, even between the forelimbs and hindlimbs of the same animal. *Hox* genes regulate the proper growth and patterning of tetrapod limbs. In order to evaluate whether variations in the complex regulation of a cluster of *Hox* genes—the *Hoxd* genes—during limb development contribute to the differences in limb shape, we compared their transcriptional control during limb bud development in the forelimbs and hindlimbs of mouse and chicken embryos. We found that the regulatory mechanism underlying *Hoxd* gene expression is highly conserved, but some clear differences exist. For instance, we observed a variation in the topologically associating domain (TAD; a self-interacting genomic region) boundary interval between the mouse and the chick, as well as differences in the activity of a conserved enhancer element situated within the telomeric regulatory domain. In contrast to the mouse, the chicken enhancer has a stronger activity in the forelimb buds than in the hindlimb buds, which is correlated with the striking differences in the mRNA levels of the genes. We conclude that differences in both the timing and duration of TAD activities and in the width of their boundary may parallel the important decrease in *Hoxd* gene transcription in chick hindlimb buds versus forelimb buds. These differences may also account for the slightly distinct regulatory strategies implemented by mammals and birds at this locus.

## Introduction

Tetrapod limbs are organized into three parts bearing skeletal elements—the stylopodium (humerus/femur), the zeugopodium (radius/fibula, ulna/tibia), and the autopodium, the latter including the acropod (phalanges, metacarpals/metatarsals) and the mesopodium (carpals and tarsals) [1]. Limbs can display large variations in their morphologies—either between tetrapod species or within the same species—as a result of their adaptation to different functions and ecological niches. For example, frogs display particular shapes of carpal and tarsal elements, with an elongated proximal tarsal whenever detectable [2], whereas geckos’ forelimb skeletal elements resemble those of their hindlimbs [3]. Another example of this morphological flexibility are the forelimbs of bats, which have digits early on similar to those of other mammals but that subsequently elongate to make flight possible [4].

In this context, birds are a fascinating taxon, as they evolved forelimbs (wings) and hindlimbs (legs) specialized for flying or for terrestrial locomotion, respectively [5]. Recent studies using comparative genomics approaches either amongst birds or between bats and mice have revealed that some bat or bird DNA enhancer sequences potentially involved in limb development and highly conserved can display differential enhancer activities as compared to their mouse orthologous sequences [6,7]. Furthermore, the analysis of several domestic pigeons displaying variations in foot feathering within the same species has suggested that changes in *cis*-regulatory elements in the genes encoding forelimb- or hindlimb-specific transcription factors may contribute to a partial transformation from hindlimb to forelimb identity [8]. Taken together, these observations indicate that both the gain of species-specific enhancers and the different activities of the same regulatory sequences, as well as alterations in DNA sequences amongst various species and/or within the same species, contributed to generate these important morphological differences.

In addition to their essential role during axial patterning and organogenesis in vertebrates [9,10], *Hox* genes are required for proper growth and skeletal patterning of tetrapod limbs. In particular, genes belonging to the *HoxA* and *HoxD* clusters are necessary for both fore- and hindlimb development. In addition, some genes of the *HoxC* cluster contribute to hindlimb development only [11,12]. In the case of both the *HoxD* and *HoxA* cluster genes, chromosome conformation techniques have made it possible to associate previously defined limb regulatory landscapes to large chromatin interaction domains referred to as topologically associating domains (TADs) [13–15]. Therefore, multiple limb-specific enhancers were identified on either side of the *HoxA* and *HoxD* clusters belonging to distinct TADs [16–19].

At the murine *HoxD* locus, two partially overlapping subsets of genes are controlled by a series of enhancers located in the corresponding TADs, located either on the telomeric side (telomeric regulatory domain [T-DOM]) or on the centromeric side (centromeric regulatory domain [C-DOM]) of the cluster [17]. The region of the cluster extending from *Hoxd1* to *Hoxd8* generates constitutive interactions with T-DOM, whereas the 5′ region of the cluster, which includes *Hoxd13* to *Hoxd12*, predominantly contacts C-DOM. The *Hoxd9* to *Hoxd11* genes interact first with T-DOM in proximal cells and subsequently with C-DOM in distal cells, and hence, they are transcribed in both the future proximal and distal domains. After an initial expression of *Hoxd1* to *Hoxd11* in the prospective zeugopod controlled by enhancer elements situated in T-DOM, *Hoxd9* to *Hoxd11* switch to establish interactions within C-DOM-located enhancers, along with *Hoxd12* and *Hoxd13*, in cells making the autopod. This switch is partly controlled by HOX13 proteins, which inhibit T-DOM activity while reinforcing C-DOM-located enhancers’ function [20]. This bimodal regulatory mechanism allows the production of a cellular domain of low *Hoxd* expression in which both T-DOM and C-DOM regulations are silent, giving rise to the future wrist and ankle articulations. Although this complex system seems to be globally conserved throughout evolution [21,22], some modifications thereof could have led to important changes in the distribution of the expression domains.

The morphological diversifications seen amongst tetrapods between fore- and hindlimbs, in particular in the mesopod and the zeugopod, were suggested to result partly from variations in *Hox* gene expression, either through gain or loss of function [2,23]. For instance, the ectopic expression of *Hoxa13* and *Hoxd13* in the proximal limb domain induces a substantial reduction and malformation of the zeugopod, similar to mesomelic dysplasia conditions in human families (e.g., [24]). This is due to the potential of these particular HOX13 proteins to antagonize the function of other HOX proteins to control and stimulate the ossification of limb skeletal elements [25]. In this view, the production of HOX protein controlled by the T-DOM (e.g., HOXD10, HOXD11) would stimulate bone growth, whereas C-DOM enhancers up-regulate *Hoxd13* to antagonize this property, leading to both smaller bones (phalanges) and the termination of the structure, in a dose-dependent manner [26–30].

In this context, a bat regulatory sequence located within T-DOM and controlling *Hoxd* genes was recently shown to display differential enhancer activity in the limbs when compared to its mouse orthologous sequence [6], supporting the idea that changes in limb morphology may rely upon variations of the bimodal gene regulation mechanism described at the *HoxD* locus. Thus far, this mechanism has been analyzed only during the development of forelimb buds. Therefore, it remains unclear how much regulatory variation, if any, may be scored between fore- and hindlimbs of the same species or between different ones.

**Fig 1:**
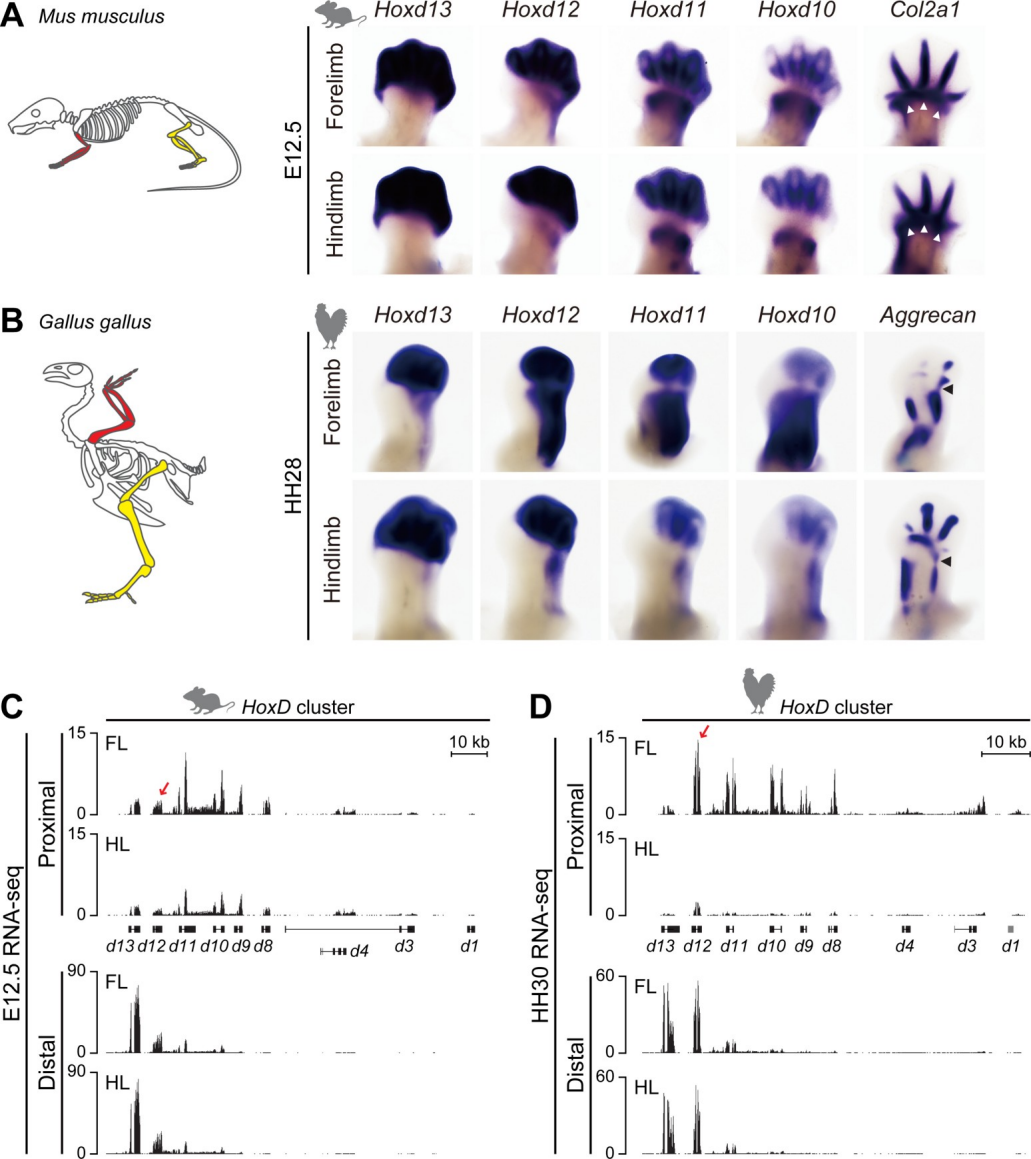
*Hoxd* gene expression in mouse and chick limb buds. (A, B) WISH analysis of E12.5 mouse and HH28 (equivalent to E12.25 to E12.5) chick FL and HL buds with expression of *Hoxd* gene and *Col2a1* or *Aggrecan*, which are markers for chondrocyte differentiation. (A, left) Schemes showing the morphologies of FL (red) and HL (yellow) in adult mice. (A, right) Expression of *Hoxd* gene in FL buds are comparable to those in HL buds. The expression domain of *Col2a1* (white arrowheads) corresponds to a low-*Hoxd*-expression region leading to the future mesopodium. (B, left) Schemes representing morphologies of FL (red) and HL (yellow) buds in chicken. (B, right) Expression of *Hoxd* gene in proximal HL is significantly reduced and restricted to the presumptive fibula. (C, D) RNA-seq profiles of *Hoxd* gene in microdissected proximal and distal domains from either E12.5 mouse (C) or HH30 (equivalent to E13 to E13.5) chick (D) FL and HL buds. Expression level of *Hoxd12* was slightly stronger in mouse proximal FL than in proximal HL (red arrow in C), a difference more pronounced in chick (red arrow in D). Right limbs in (A, B) are oriented proximally to the bottom and distally to the top. The *y* axis represents the strand-specific RNA-seq read counts, normalized by the total number of million mapped reads. *Col2a1*, *collagen type II alpha 1 chain gene*; E, embryonic day; FL, forelimb; HH, Hamburger–Hamilton stage; HL, hindlimb; RNA-seq, RNA sequencing; WISH, whole-mount in situ hybridization.

To tackle this issue, we used a comparative regulatory approach involving chick and mouse embryonic fore- and hindlimbs, mostly for two reasons. First, chicken embryos, unlike mice, display striking differences between the morphologies of their adult forelimbs and hindlimbs (Fig 1A and 1B, left). Second, it was reported that *Hoxd* gene expression domains during chick fore- and hindlimb buds’ development showed important deviations when compared to their mouse counterparts [23,31]. These features suggested that the bimodal regulatory system at work at the mouse *HoxD* locus may be operating slightly differently during the development of the avian appendicular skeletons.

Here, we combine the analyses of transcriptome, 3D genome conformation, histone modification, and mouse genetics to show that this bimodal regulatory mechanism is highly conserved in birds. However, in chicken hindlimb buds, the duration of T-DOM regulation is importantly shortened, which accounts for the concurrent reduction in *Hoxd* gene expression in the zeugopod. By using mutant mouse embryos lacking a large part of T-DOM, we also uncovered regulatory differences between fore- and hindlimbs. Therefore, although the general principles of these regulatory mechanisms are similar either amongst tetrapod species or within the same species between the fore- and hindlimbs, slight differences are scored, which may partly contribute to the observed morphological differences.

## Results

### Transcription of *Hoxd* genes in mouse and chick limb buds

We first used whole-mount in situ hybridization (WISH) to compare the expression patterns of *Hoxd* genes in mouse fore- and hindlimbs at embryonic day (E)12.5 (Fig 1A) with those observed in chick at either Hamburger–Hamilton stage (HH)28 (equivalent to E12.25–E12.5, see also S1 Fig) (Fig 1B) or HH30 (equivalent to E13–E13.5, see also S1 Fig). In mouse fore- and hindlimbs, the amounts of *Hoxd13* and *Hoxd12* mRNAs were high in the prospective acropod region (hereafter termed “distal”), whereas *Hoxd11* and *Hoxd10* transcripts were detected in both the distal and zeugopod (hereafter termed “proximal”) regions, separated by the future mesopodial articulation, which was labeled by the *collagen type II alpha 1 chain gene* (*Col2a1*; Fig 1A, arrowheads). These expression patterns were similar in both fore- and hindlimbs, except for a clearly weaker expression level in the hindlimb proximal domain.

When compared to the corresponding mouse expression patterns, at least two salient differences were confirmed. First, unlike the *Hoxd12* expression pattern observed in murine limbs, the chick *Hoxd12* gene was strongly expressed in proximal forelimb (Fig 1B). Second, the expression of all *Hoxd* genes was significantly reduced in the chick proximal hindlimb by stage HH28, when compared to both chick proximal forelimb and mouse proximal limbs [23,31]. As a result, the expression domains of the chick *Hoxd12* in forelimb buds appeared much like that of *Hoxd11* or *Hoxd10* in contrast to the mouse, in which *Hoxd12* is only very weakly expressed in proximal cells. However, the transition between the two *Hoxd-*expressing domains also labeled the future forelimb mesopod (Fig 1B, arrowheads). Of note, expression of all *Hoxd* genes was weak in proximal hindlimb buds, again in contrast to what was observed during mouse limb bud development (Fig 1).

To further characterize these differences, we performed RNA sequencing (RNA-seq) analyses by using HH30 limb buds in order to more precisely microdissect the various domains and thus exclude any potential contamination of the future mesopod from the distal domain. RNA-seq profiles confirmed the differences detected by WISH. First, *Hoxd11* to *Hoxd8* were expressed at lower levels in the mouse proximal hindlimb when compared to forelimb (Fig 1C, upper tracks), with Fragments Per Kilobase of exon model per Million mapped fragments (FPKM) values decreased about 2-fold (S1 Table). This situation is reinforced in chick proximal hindlimb, in which *Hoxd8* to *Hoxd11* are nearly not expressed (values of FPKM below 5 for proximal hindlimb, compare to above 30 for proximal forelimb; S1 Table, see also Fig 1D, upper tracks). *Hoxd12* expression was higher in proximal hindlimb but still lower than in proximal forelimb. In contrast, more reads were scored for *Hoxa10* to *Hoxa11* in both mouse and chick proximal hindlimb when compared to forelimb (S1G Fig and S1 Table).

In the distal domains, transcription patterns and profiles from mouse and chick were similar between fore- and hindlimbs for both the *HoxA* and *HoxD* clusters (Fig 1C and 1D, lower tracks, S1F and S1G Fig). However, the chick profile revealed a higher transcription of *Hoxd12*. In distal limbs, *Hoxd12* expression was higher than *Hoxd13* in chick, whereas the FPKM values in the mouse counterpart were about one-third of those for *Hoxd13* (Fig 1C and 1D, lower tracks and S1 Table). In chicken proximal limbs, *Hoxd12* expression was about 10-fold higher than *Hoxd13*, whereas in mouse these two genes are in the same range (Fig 1C and 1D, upper tracks red arrow and S1 Table). Taken together, these initial results indicated that both the expression quantities and transcript domains of *Hoxd* genes displayed significant differences, either between species or the developing fore- and hindlimb buds. This was particularly evident in chicken.

### Bimodal regulation in both fore- and hindlimb buds

To determine to what extent these differences could result from variations in the implementation of the bimodal regulatory mechanism, we performed comparative circular chromosome conformation capture (4C) sequencing (4C-seq) analyses. We used a variety of 4C viewpoints located at comparable positions to reveal potential interactions in both mouse and chicken limb buds. To do this, we cross-annotated those *Hoxd* genes’ regulatory sequences identified in the mouse genome onto the chicken genome by using the LiftOver tool in the University of California, Santa Cruz (UCSC) genome browser. These annotations were then used for all following experiments. In both fore- and hindlimbs, interactions were scored between *Hoxd* genes and the regulatory sequences *island III* and *Prox*, which are hallmarks of C-DOM transcriptional activity. Alternatively, interactions scored with the *CS39* sequence were used as a proxy for T-DOM activity in the distal and the proximal regions, respectively [17,18].

**Fig 2:**
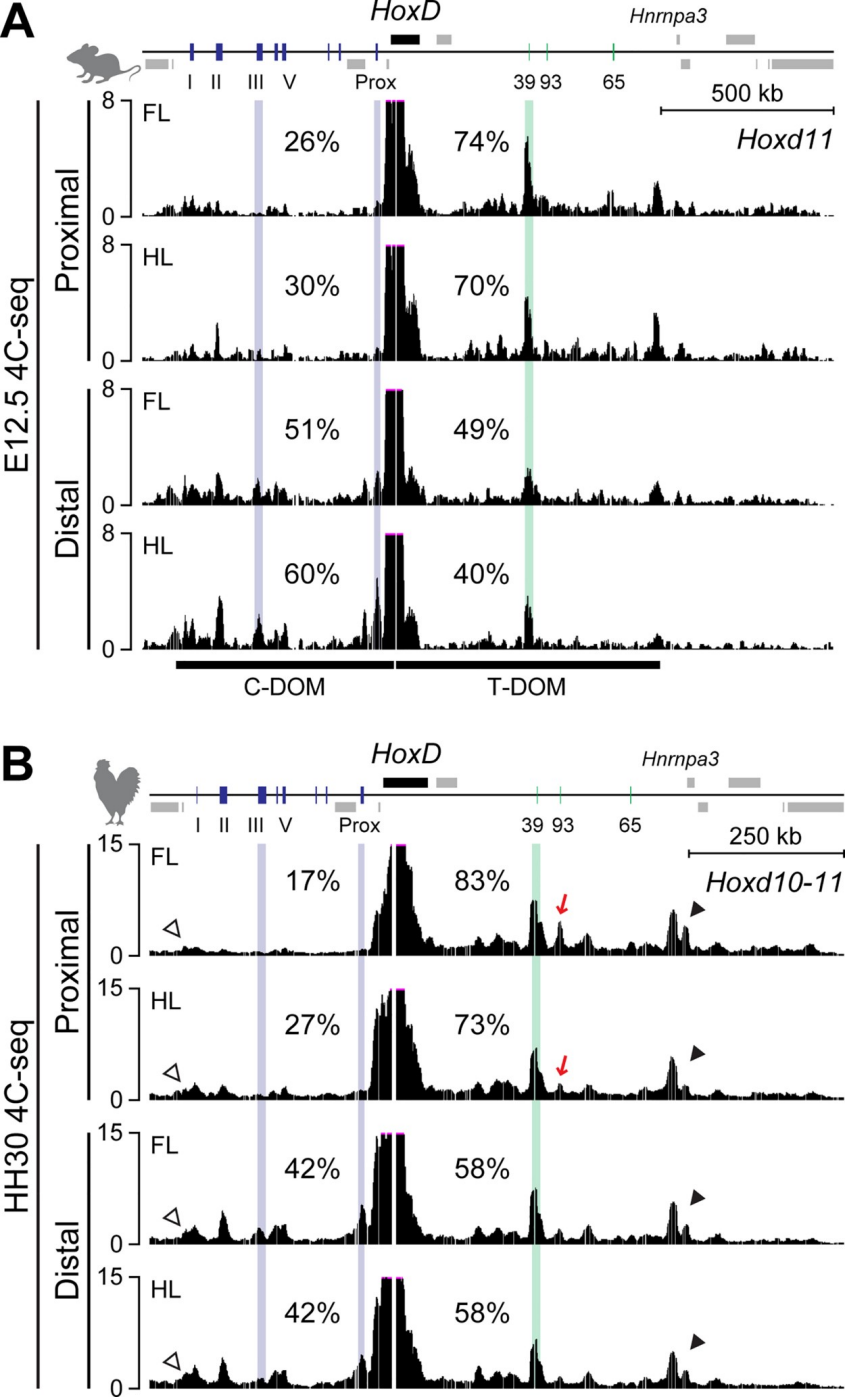
Conserved bimodal regulation at the chick *HoxD* locus. (A, B) 4C-seq tracks showing contacts established by mouse *Hoxd11* (A) and chick *Hoxd10-11* (B) viewpoints in mouse and chick proximal and distal cells from FL and HL at E12.5 and HH30, respectively. (A) The interactions between *Hoxd11* to and around the *CS39* region were mainly observed in proximal cells, whereas those between *Hoxd11* and either *island III* or *Prox*, which are hallmarks of the C-DOM activity, were increased in the distal region. (B) The contacts extend up to the predicted borders of the two TADs located on either side of the *HoxD* cluster (C-DOM, opened arrowheads; T-DOM, closed arrowheads). In addition to the interactions between *Hoxd10-11* and *CS39*, contacts were also observed with *CS93* in proximal FL bud cells. These contacts are decreased in proximal HL bud cells in which *Hoxd* expression is strongly reduced (red arrows). 4C-seq, circular chromosome conformation capture sequencing; C-DOM, centromeric regulatory domain; E, embryonic day; FL, forelimb; HH, Hamburger–Hamilton stage; HL, hindlimb; TAD, topologically associating domain; T-DOM, telomeric regulatory domain.

As seen in mouse forelimbs, *Hoxd11* mainly contacted *CS39* and other T-DOM sequences in mouse proximal hindlimb cells, i.e., in cells in which T-DOM was fully active and in which C-DOM was silent (Fig 2A, top). In contrast, in mouse distal hindlimb cells, *Hoxd11* preferentially interacted with C-DOM sequences such as *island III* and *Prox* (Fig 2A, bottom). Quantification of contacts indicated 74% of telomeric contacts in proximal forelimb cells and 49% in distal forelimb cells, showing that *Hoxd11* had reallocated 25% of its global interactions toward the C-DOM TAD in distal cells. Likewise, mouse hindlimb cells showed the same interaction profiles, with 70% of telomeric contacts in proximal hindlimb cells and 40% in distal hindlimb cells (Fig 2A). This comparison indicated that the bimodal regulation is similar between fore- and hindlimbs in mouse.

We then examined these interaction patterns in chick fore- and hindlimb cells by using a region between *Hoxd11* and *Hoxd10* as a viewpoint (termed *Hoxd10-11*), i.e., a sequence located as close as possible to the bait used in the mouse experiments. In chick proximal forelimb cells, *Hoxd10-11* interacted mostly with the *CS39* and *CS93* regions located in T-DOM, as well as with a region near the *Hnrnpa3* gene at which the distal TAD border is observed in the murine locus (Fig 2B, black arrowhead). Each of these predominant contacts with T-DOM were reduced by 2% to 5% in chick distal forelimb cells: 14% to 11% for *CS39* (*p*-value = 2e-3), 8% to 3% for *CS93* (*p*-value = 2e-7), and 6% to 4% for the TAD border (*p*-value = 3e-3). As in the mouse, 25% of contacts were indeed reallocated to C-DOM sequences such as the chicken *island III* (+3%, *p*-value = 1e-8) and *Prox* (+6%, *p*-value = 3e-8) sequences. When compared to chick proximal forelimb cells, the global interaction with the T-DOM was decreased from 83% to 73% in proximal hindlimb cells. In particular, the interactions between the *Hoxd10-11* bait and the *CS93* sequence in T-DOM were decreased in proximal hindlimb cells (from 8% to 4%, *p*-value = 3e-5), which may account for the significant reduction of *Hoxd* expression in chick proximal hindlimb buds (Fig 2B, red arrows). In contrast, the interaction established by the *Hoxd10-11* bait in chick fore- and hindlimb distal cells were comparable (maximum 1% difference in all quantified regions and *p*-values above 0.05), as expected from transcripts analyses, and interactions were observed up to the vicinity of the *Atp5g3* gene where the border of C-DOM TAD has been mapped in mouse (Fig 2B, white arrowheads).

### Different enhancer activity of mouse and chick *CS93* in fore- versus hindlimbs

**Fig 3:**
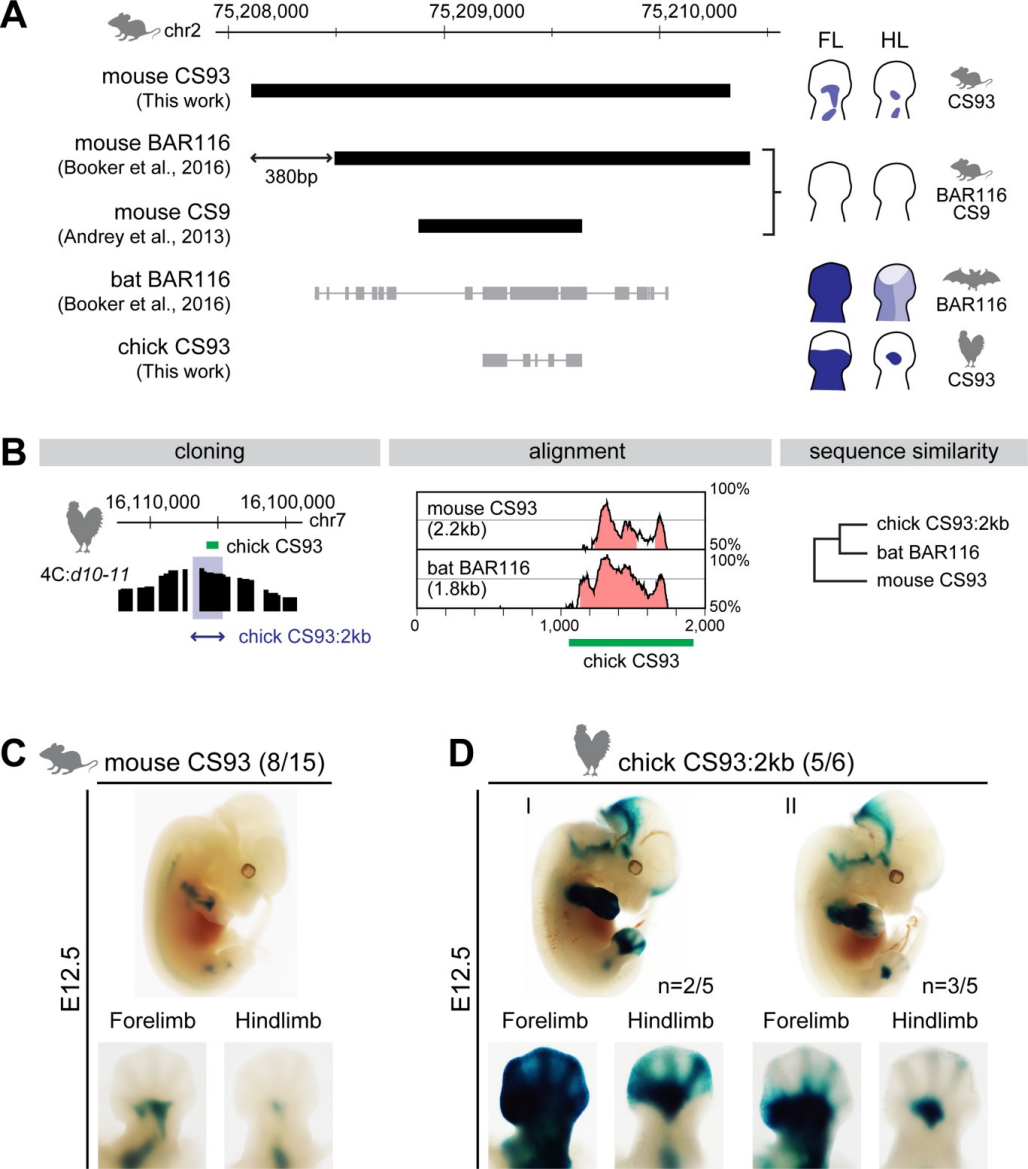
Differential enhancer activities of mouse and chick *CS93* in FL and HL buds. (A) Genomic coordinates and sequence alignment using either the bat or the chick sequence onto the mouse genome and schematics summarizing the enhancer activities for each of the identified sequences [6,17] (this work). Neither murine BAR116 nor *CS9* showed any enhancer activity in limbs [6,17], whereas the bat BAR116 displayed different patterns between mouse FL and HL [6]. The sequences of both the bat BAR116 (Myoluc2, GL429772: 6,606,808–6,608,652) and the chick *CS93* (galGal5, chr7:16,104,952–16,105,803) were aligned with BLAT onto the mouse genome. (B) (Left) Genomic coordinates of either the chick *CS93* (green rectangle) or the chick 2-kb region used in the enhancer assay (blue domain). The 2-kb sequence contains the chick *CS93* region and the region of high interactions with the *Hoxd10* to *Hoxd11* region in proximal FL bud cells at HH30. (Middle) Conservation plot of mouse *CS93* and bat BAR116 using the 2-kb region of chick *CS93* as a reference. The peaks represent a conservation higher than 50%. Pink regions are conserved noncoding sequences. (Right) The sequence similarity obtained from mVista tools shows the highest conservation of the chick *CS93* with the bat BAR116 sequences. (C, D) Enhancer activities of mouse *CS93* (C) and the 2-kb region of chick *CS93* (D) in mouse FL and HL buds E12.5. The *lacZ* expression pattern (C) showed that mouse *CS93* has an enhancer activity in the proximal region of developing limb buds at E12.5. In contrast to the mouse, the 2-kb region of chick *CS93* (D) showed differential enhancer activity between FL and HL buds at E12.5, as was also reported for the bat BAR116 sequence. The numbers of *lacZ*-positive embryos over total transgene integrated are indicated. BAR116, Bat Accelerated Region 116; E, embryonic day; FL, forelimb; HH, Hamburger–Hamilton stage; HL, hindlimb.

The mouse *CS93* sequence contains the former *CS9* sequence [17], which was reported not to elicit any reporter gene expression in a mouse transgenic context (Fig 3A). Likewise, a larger murine sequence encompassing *CS9* and referred to as mouse Bat Accelerated Region 116 (BAR116) did not show any enhancer activity in the limbs [6] (Fig 3A). In contrast, the corresponding bat BAR116 sequence was able to drive strong expression in transgenic mouse forelimb buds, whereas only a weak activity was detected in hindlimb buds, correlating with the different expression levels of *Hoxd10* and *Hoxd11* observed between bat fore- and hindlimb buds [6] (Fig 3A). This sequence was thus proposed as having evolved a “bat-specific” function.

Since the low expression of *Hoxd* genes in proximal hindlimbs seems to be a common feature of bats and chicken, we hypothesized that the chick *CS93* sequence may have a limb enhancer activity similar to that reported for the bat BAR116. We examined the enhancer activity of chick *CS93* using a transgenic mouse *lacZ* reporter system and compared it to the activity of full-length mouse *CS93* sequence by using lentivector-mediated transgenesis [32,33]. We initially cloned a 2-kb sequence containing chick *CS93* and more surrounding sequences (Fig 3B), which showed higher interactions with *Hoxd10* to *Hoxd11* in the 4C profiles obtained from proximal forelimb cells (Fig 2B, track 1). We noted that the surrounding sequences are not particularly conserved among these species, whereas the *CS93* region of the chick genome is more conserved with the bat than with the mouse counterpart (430 bp 88% identity and 234 bp 89% identity, respectively; Fig 3B and S2 Fig). By using the BLAT search tool in UCSC, we also found that most of the conserved regions from the bat BAR116 and the chick *CS93* sequences can be aligned onto the mouse *CS9* region (Fig 3A).

We assessed their enhancer activities and, unlike for the mouse BAR116, the full-length mouse *CS93* triggered *lacZ* transcription in E10.5 limb buds with an expression localized to the prospective stylopod and zeugopod at E12.5 (Fig 3C and S2B Fig). The staining was weaker in hindlimb than in forelimb buds, possibly because of the delay in limb development [34,35]. Accordingly, the 380 bp localized in 5′ of the mouse *CS93* seemed to be essential for expression. On the other hand, we found that the 2-kb sequence containing the chick *CS93* displayed limb enhancer activity in mouse limb buds at E12.5 (Fig 3D and S2C Fig). The reporter transgene driven by chick *CS93* generated two different patterns. The first one displayed *lacZ* staining throughout the forelimbs (*n* = 2/5), which was similar to the staining observed when the bat BAR116 sequence was assessed in mouse forelimb bud (Fig 3D, S2C Fig, and Fig 4 in [6]). In the second pattern, most of the staining was observed in the proximal forelimb buds (*n* = 3/5), as seen when the mouse *CS93* was used (Fig 3C). In both cases, a weaker expression was observed in hindlimb bud, as in the case of bat BAR116. These results suggest that the down-regulation of *Hoxd* genes in chick hindlimb bud is associated with a generally weaker activity of—and fewer interactions with—the *CS93* sequence.

### Implementation of the regulatory switch between TADs in mouse and chicken

The differences observed in *Hoxd12* expression, in particular between mouse and chick proximal forelimbs (Fig 1), raised the possibility that the regulatory switch from T-DOM to C-DOM enhancers would be implemented in a slightly different manner in the two species. We thus produced and examined 4C interaction profiles by using *Hoxd12* itself as bait. Similar to the profiles obtained with the *Hoxd10-11* bait, we observed weaker interactions between *Hoxd12* and both the *CS39* and *CS93* regions in T-DOM in chick proximal hindlimb cells than in proximal forelimb cells, from 12% to 9% for *CS39* (*p*-value = 4e-3) and from 5% to 3% for *CS93* (*p*-value = 4e-3) (S3A Fig, top red arrows). The profiles with the *Hoxd10-11* bait showed strong and stable interactions with T-DOM, when compared with C-DOM, in both proximal and distal limbs (Fig 2B). We also found that *Hoxd12* mainly contacted T-DOM in both chick proximal fore- and hindlimb cells (60% to 63%), whereas it established more interactions with C-DOM in both chick distal cells (62% to 64%; S3A Fig bottom).

The murine *Hoxd9* to *Hoxd11* genes, but not *Hoxd12*, are located in the region of the TAD boundary and interact both with T-DOM and with C-DOM. In contrast, in chicken limb buds, *Hoxd12* was able to switch contacts from T-DOM to C-DOM, suggesting that the TAD boundary in chick could be located at a more centromeric position, between *Hoxd12* and *Hoxd13* (see also S3B and S3C Fig), whereas this switch region was localized around the *Hoxd11* locus in the mouse [17,19]. This same switch was observed in both chick fore- and hindlimb bud cells, regardless of the various expression levels of *Hoxd* genes in the proximal region, indicating that the switch between TADs is independent of *Hoxd* gene expression in proximal cells while dependent on *Hoxa13* and *Hoxd13* expression in distal cells [20].

These results showed that the bimodal regulatory mechanism and the sequential transition from the proximal to the distal global controls are implemented during chick limb development similarly to what was described in mice. Therefore, the differences in gene expression observed both between mice and chicken and between chick fore- and hindlimb buds cannot be solely explained by visible variations in the respective interaction profiles. Instead, they ought to involve the distinct use of enhancers (or groups thereof) within an otherwise globally conserved chromatin architecture.

### Premature termination of the telomeric TAD activity in chick hindlimb buds

**Fig 4:**
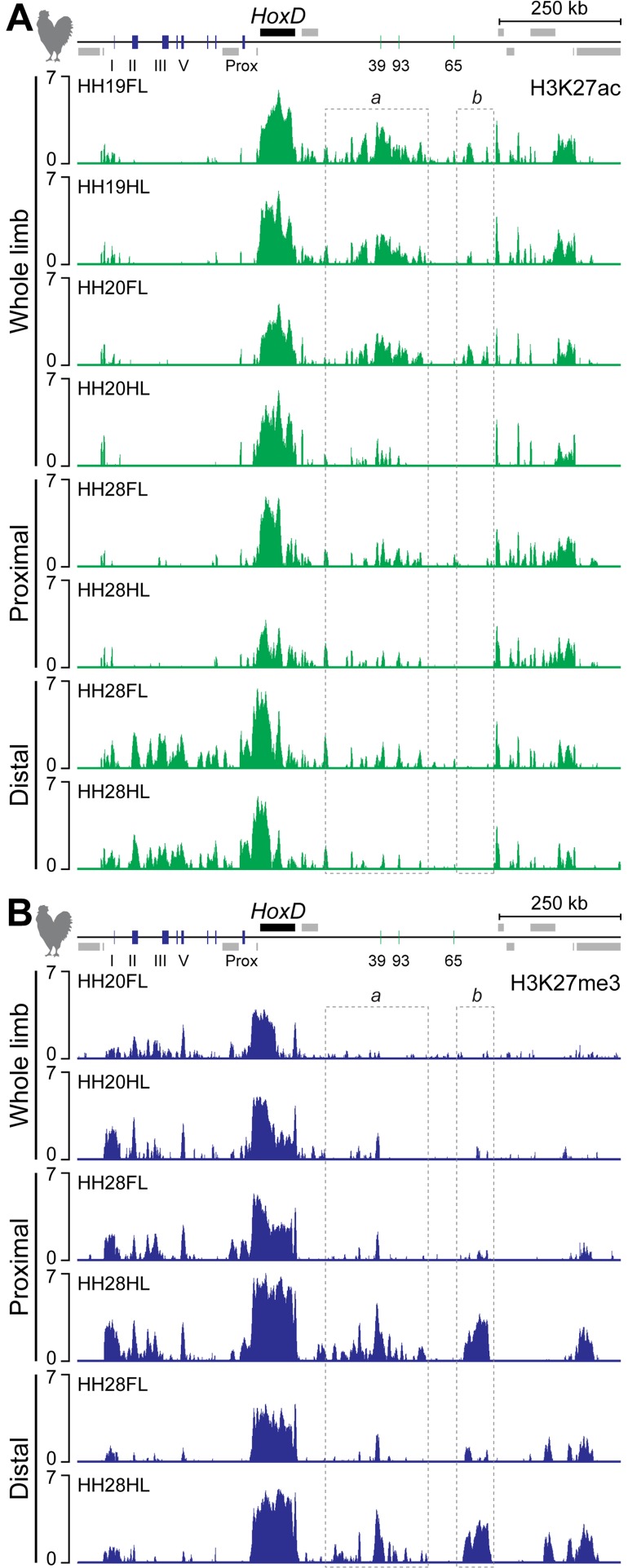
Premature termination of T-DOM activity in chick HL buds. (A, B) Comparison of H3K27ac and H3K27me3 ChIP-seq profiles in either whole, proximal, or distal FL and HL buds at HH19 (equivalent to mouse E9.5), HH20 (equivalent to mouse E10), and HH28. (A) In chick HL bud, enrichment of H3K27ac at region a in T-DOM was initially detected at HH19, whereas it was significantly decreased at HH20. Few H3K27ac marks were scored in region b in HL bud at both HH19 and HH20, as compared with those in FL buds. At HH28, the accumulation of H3K27ac marks was quite low in both the *HoxD* cluster and the T-DOM region in proximal HL when compared to distal FL cells, whereas the profiles of H3K27ac in the distal region where *Hoxd* genes are strongly expressed were similar between FL and HL buds at HH28. (B) In FL and HL buds at HH20, several C-DOM regions were decorated by H3K27me3. In contrast, T-DOM was not labeled in FL buds at this stage, nor had H3K27me3 marks started to accumulate around *CS39* in HL buds. In proximal HL buds where *Hoxd* expression was reduced, H3K27me3 enrichment was observed at the *HoxD* cluster and over T-DOM when compared to proximal FL buds. Both regions a and b in T-DOM were enriched in H3K27me3. Enrichment (*y* axis) of ChIP is shown as the log2 ratio of the normalized number of reads between ChIP and input samples. C-DOM, centromeric regulatory domain; ChIP, chromatin immunoprecipitation; ChIP-seq, ChIP sequencing; FL, forelimb; H3K27ac, acetylation of histone H3 lysine 27; H3K27me3, trimethylation of H2K27; HH, Hamburger–Hamilton stage; HL, hindlimb; T-DOM, telomeric regulatory domain.

Since the chromatin architecture at the *HoxD* locus is seemingly comparable between mouse and chicken in both fore- and hindlimb buds, we looked for what may cause the drastic reduction of *Hoxd* expression observed in chick proximal hindlimb. Within the T-DOM TAD structure, the interaction profiles obtained from chick hindlimb proximal cells showed reduced contacts between *Hoxd* promoters and enhancers in T-DOM (Fig 2 and S3 Fig). We complemented these observations by assessing the functional state of T-DOM sequences by comparing particular histone modifications profiles between chick fore- and hindlimb buds at several developmental stages (Fig 4). We looked at the acetylation of histone H3 lysine 27 (H3K27ac), a modification associated with transcriptional and enhancer activity, and at the trimethylation of H3K27 (H3K27me3), a mark associated with Polycomb-dependent silencing [36]. In both fore- and hindlimb buds at stage HH19, a stage that corresponds to about E9.5 in mouse, enrichments of H3K27ac were detected over both T-DOM and the *HoxD* cluster itself, showing that the activation of *Hoxd* genes by the T-DOM enhancers had been properly initiated in hindlimb buds (Fig 4A, tracks 1 and 2). Of note, higher levels of this mark were scored over the *Hoxd11* to *Hoxd13* region in hindlimb than in forelimb buds, with an enrichment from 3 to 4 over this region (S1 Table), whereas it remained stable over the rest of the cluster (S4A Fig).

At stage HH20 (approximatively E10 in mouse), the H3K27ac enrichment in T-DOM was still substantial in forelimb buds (enrichment of 0.9 in region a and of 0.9 to 1.2 at stage HH19). In marked contrast, however, this level appeared dramatically reduced in hindlimb buds (no enrichment, Fig 4A, tracks 3 and 4, region a), thus coinciding with low gene expression (S4A Fig tracks 3 and 4). The accumulation of H3K27ac observed near the distal TAD border was specific for the early forelimb bud (enrichment of 0.4 to 0.5 in HH19 and HH20 forelimbs, whereas below −0.3 in other conditions; Fig 4A, tracks 1 and 3, region b). Furthermore, H3K27ac signals over C-DOM were not yet observed at these stages (except around the *island I* region), in agreement with the fact that the regulatory switch had not yet occurred (enrichment over C-DOM below −0.6). At a later stage (HH28, the equivalent of approximately E12.5 in mouse), enrichment of H3K27ac within the *HoxD* cluster was significantly lost in proximal hindlimb bud cells where *Hoxd* expression was weak (enrichment of 1.5, whereas above 2.7 in all other conditions; Fig 4A, track 6; S4A Fig, track 8). In contrast, H3K27ac accumulation over the T-DOM in proximal forelimb bud cells remained, yet it started to slowly decrease, as observed in mouse proximal forelimb at E12.5 (enrichment of 0.3 in both proximal and distal forelimbs while negative in hindlimb tissues; Fig 4A, track 5 and 7). At the same time, H3K27ac was finally enriched over both C-DOM and the *HoxD* cluster in both fore- and hindlimb distal cells (enrichment over 1 in C-DOM, in contrast to values below −0.5 for other tracks), as scored in mouse distal forelimb buds (Fig 4A, tracks 7 and 8; S4A Fig, tracks 9 and 10) [17,20]. These various profiles showed that in chick hindlimb bud cells, the functional switch between T-DOM and C-DOM had occurred normally, except that after its initial onset, T-DOM activity was terminated much more rapidly than in the forelimb bud, followed by a decrease in accumulation of H3K27ac at the target *HoxD* cluster itself.

We complemented these observations by analyzing H3K27me3 marks, which antagonize H3K27ac [36]. At stage HH20, no clear H3K27me3 signal was detected over T-DOM either in fore- or in hindlimb buds (Fig 4B, tracks 1 and 2, region a), in agreement with the H3K27ac profiles (compared with Fig 4A, tracks 3 and 4). In contrast, strong levels of H3K27me3 enrichment were observed over the C-DOM regions, where H3K27ac peaks were not detected (enrichment of 0.3; Fig 4B, tracks 1 and 2), suggesting that the activation of *Hoxd* genes by C-DOM regulation had not yet occurred at this stage.

At the *HoxD* cluster itself, stronger levels of H3K27me3 enrichment were clearly detected in hindlimb buds (as compared with forelimb buds) from the pseudo-*Hoxd1* gene to *Hoxd8* (enrichment of 2.3, compared to 0.7), a DNA interval controlled by T-DOM regulation (S4B Fig, tracks 1 and 2). At later stages, H3K27me3 marks were observed over C-DOM in proximal forelimb bud cells (enrichment of 0.6), in which C-DOM is inactive, whereas the levels of H3K27me3 marks over T-DOM in both proximal and distal forelimb bud cells were somewhat comparable to those seen in the H3K27ac profiles (Fig 4B, tracks 3, 5).

Altogether, the distribution of both H3K27ac and H3K27me3 marks in chicken limb buds matched the observed expression profiles of *Hoxd* genes. A major difference was scored, however, when compared to their mouse counterparts. In proximal hindlimb bud cells at HH28, in which *Hoxd* gene expression is quite weak, T-DOM and the *HoxD* cluster were heavily decorated with H3K27me3 marks (enrichment of 1 for region a, of 2.3 for region b, and of 5.4 for the *HoxD* cluster), in addition to the C-DOM TAD (enrichment of 0.8; Fig 4B, track 4). The profile over T-DOM resembled that obtained from distal hindlimb bud cells at the same stage—i.e., cells in which T-DOM is inactive and completely shut down (Fig 4B, track 6; regions a and b, enrichment of 0.7 and 2.1, respectively). This suggests that T-DOM was not operational in proximal hindlimb cells at this stage, unlike in the mouse forelimb proximal situation [17,20].

We also examined the distribution of both H3K27ac and H3K27me3 marks over the *HoxA* cluster and its limb regulatory landscape that maps within a sub-TAD adjacent to *Hoxa13* [16] (S5 Fig). Qualitatively, H3K27ac enrichments in this regulatory landscape were fairly similar between fore- and hindlimb tissues at all stages examined (S5A Fig). However, at the level of single enhancers, we detected differences in enrichment between fore- and hindlimb buds (see S1 Table).

### Chromatin conformation of the chick *HoxD* cluster in fore- and hindlimb buds

Gene expression often occurs concomitantly with enhancer–promoter contacts [37–39]. Because of the dramatic difference in T-DOM activity observed in chick hind- versus forelimb buds at stage HH20 (Fig 4A, tracks 3 and 4, region a), we looked for potentially related differences in chromatin contacts by performing high-resolution with high-throughput chromosome conformation capture (capture Hi-C [CHi-C]) technology [40,41] using fore- and hindlimb buds at HH20. Since such a global chromatin assessment had not been evaluated during chick development, it also allowed us to compare it with mouse counterpart cells and see to what extent these complex regulatory systems were conserved in distinct groups of tetrapods.

**Fig 5:**
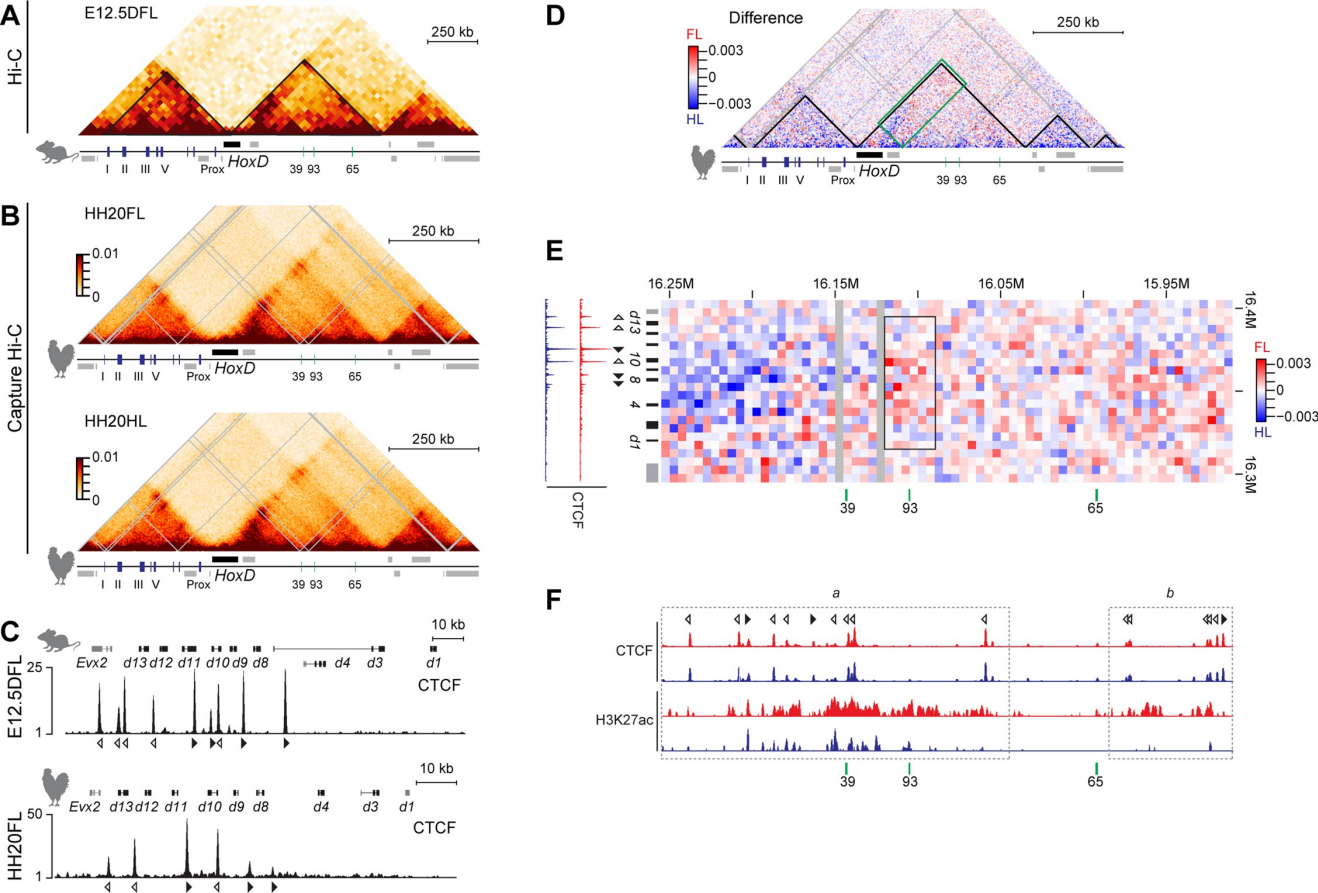
Chromatin conformation and bound CTCF sites at the mouse and chick *HoxD* locus. (A) Hi-C heat map data adapted from [19] at a 40-kb resolution. The black lines demarcate the TADs in mouse distal FLs at E12.5. (B) CHi-C heat maps at 5-kb resolution by using either chick FL or HL buds at HH20. (C) Comparison of bound CTCF and site orientations at the *HoxD* cluster between mouse distal FL at E12.5 (top) and chick FL bud at HH20 (bottom). Open and closed arrowheads indicate the orientations of the CTCF motifs. (D) Subtraction of the CHi-C matrices shown in (B), with FL bud cells in red and HL bud cells in blue. The black lines demarcate the TADs. The green rectangle is enlarged in (E). (E, F) Subtraction of the CHi-C matrices shown in (B) between the *HoxD* cluster and the area from region a to region b within T-DOM at a 5-kb resolution. A decrease in contacts is detected between the *HoxD* cluster and the *CS93* region in HLs (black rectangle in E), which corresponds to the reduction in H3K27ac levels seen in HL cells at HH20 (F). ChIP-seq profiles of CTCF and H3K27ac from FL and HL buds at HH20 are shown in red and blue, respectively. Open and closed arrowheads indicate the orientation of the CTCF motifs. Enrichments (*y* axis) of CTCF and H3K27ac ChIP are shown at the normalized 1x sequencing depth or the log2 ratio of the normalized number of reads between ChIP and input samples, respectively. CHi-C, capture Hi-C; ChIP, chromatin immunoprecipitation; ChIP-seq, ChIP sequencing; CTCF, CCCTC-binding factor; E, embryonic day; FL, forelimb; H3K27ac, acetylation histone H3 lysine 27; Hi-C, high-throughput chromosome conformation capture; HH, Hamburger–Hamilton stage; HL, hindlimb; TAD, topologically associating domain.

The CHi-C profiles of chicken cells confirmed that the chick *HoxD* cluster is positioned at the boundary between two TADs, similar to what was proposed in mouse limb bud tissues [17,19]. In addition, the two sub-TADs seen in the murine T-DOM were also observed in the chicken locus (Fig 5A and 5B). To position the boundary between the two TADs, we applied the TopDom algorithm [42], which determined the border around the *Hoxd13* locus in both fore- and hindlimb bud cells at HH20 (Fig 5D). This extended the conclusion reached after the 4C analyses that the TAD boundary region in chick was displaced toward the 5′ part of the gene cluster when compared to mouse limb bud cells [19].

In mouse limb cells, this TAD boundary falls within a region where multiple CCCTC-binding factor (CTCF) sites are occupied [43–45]. CTCF is an architectural protein that both helps defining constitutive domains of interaction and facilitates enhancer–promoter contacts [46]. We thus examined the presence of bound CTCF at the chick *HoxD* locus (Fig 5C) and surrounding TADs (S6A and S6B Fig) and found that the profiles were comparable between fore- and hindlimb buds at HH20. As for the mouse *HoxD* cluster [19], the orientations of the CTCF motifs located on either side of the TAD boundary were facing sites found in their flanking TADs, suggesting the possibility for long-range loops to be established (Fig 5C and S6 Fig, e.g., [47]). The orientation of the CTCF motifs were conserved between mouse and chick. However, we found fewer bound sites of CTCF in the chicken *HoxD* cluster than in the mouse counterpart, which could affect the strength and/or stability of the TAD boundary in chick.

When a CHi-C at 5-kb resolution was analyzed, the distribution of contacts was relatively similar between fore- and hindlimb bud cell populations (Fig 5B), despite the slightly reduced level of H3K27ac in the T-DOM and near the TAD border in hindlimb bud cells described above. However, this reduced level of H3K27ac in hindlimb bud cells around region b was associated with a decrease in contact probability with the *HoxD* cluster (*p*-value = 3e-5) (Fig 5D–5F). In T-DOM region a, where a reduction of H3K27ac was also scored in hindlimb bud cells, we observed two different patterns (Fig 5D–5F). The centromeric part of region a up to *CS39*, where several bound CTCF sites were scored, was more contacted in hindlimb bud cells (*p*-value = 2e-9), whereas the 30-kb region including *CS93* (black rectangle in Fig 5E) was less contacted (*p*-value = 1e-4). These reduced contacts between *Hoxd* genes and the surroundings of region *CS93* confirmed the analyses of 4C profiles obtained using the later stage (HH30) (Fig 2B). Moreover, the 4C profiles obtained when using the *Hoxd10-11* bait showed interactions with the 5′ region of *CS39* in both chick proximal and distal cells, suggesting that these stable contacts are associated with CTCF, as described in mouse developing limb buds [48]. The fact that bound CTCF was not detected around the *CS93* region suggests that CTCF-independent variations in enhancer–promoter interactions may participate in the important decrease in *Hoxd* gene expression levels in hindlimb bud cells.

### Regulation of T-DOM by HOXA13

We looked for a cause of the robust reduction in H3K27ac marks in chicken T-DOM and the decrease in contacts between *Hoxd* genes and the *CS93* region in hindlimb bud cells at HH20. We had previously reported that HOX13 proteins bind T-DOM-located sequences concomitant to the inactivation of this TAD. Also, the absence of HOX13 proteins leads T-DOM to continue operating even into distal cells [20,48]. Consequently, we assessed the expression dynamics of *Hoxa13* and found that this gene is expressed earlier in chick hindlimb bud than in forelimb buds [31] (FPKM values of 7 for hindlimbs and below 1 for forelimbs; S1 Table), suggesting that the timing of *Hoxa13* transcriptional activation may fix the duration of T-DOM activity during limb development.

**Fig 6:**
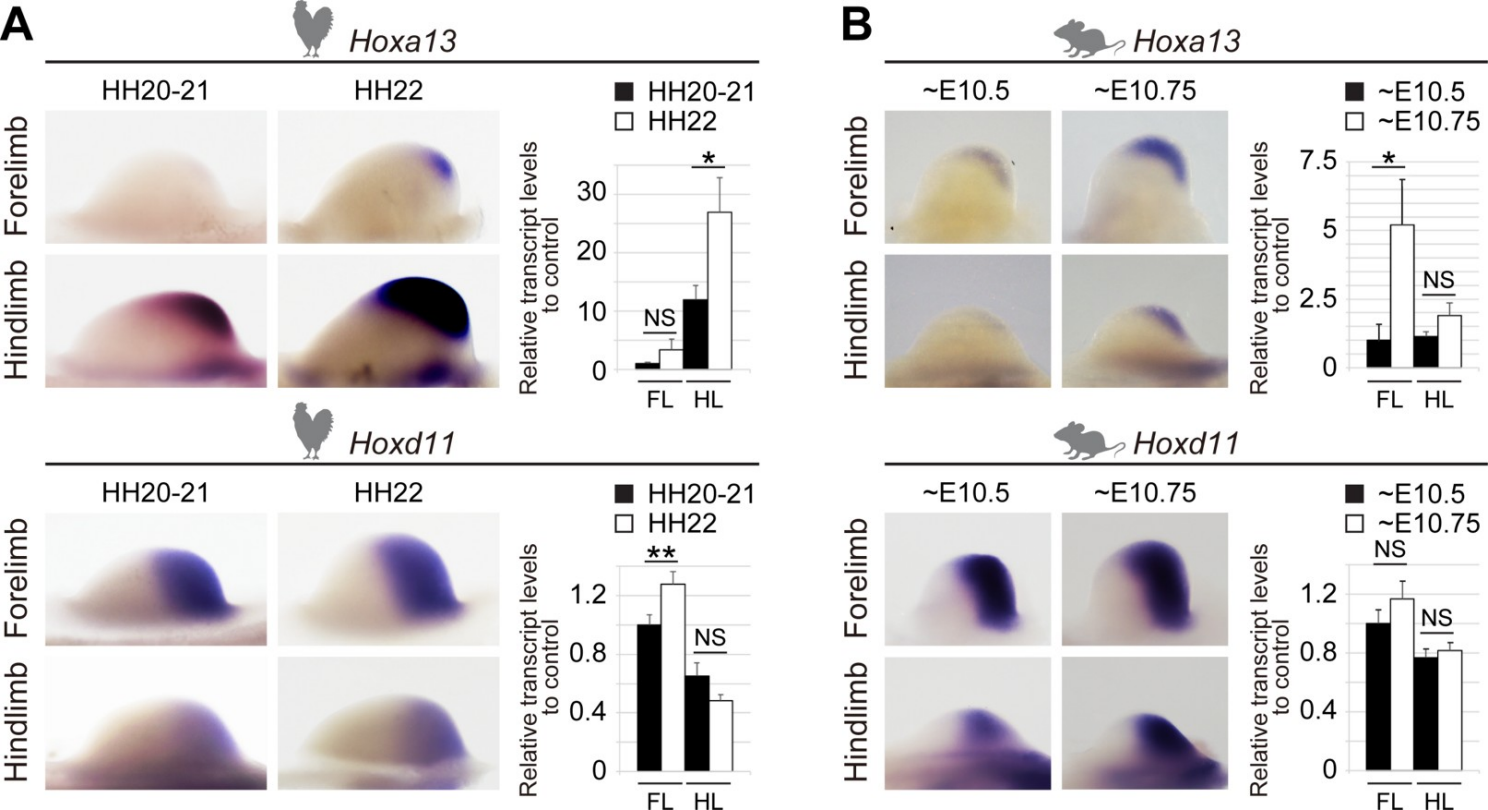
*Hoxa13* expression in chicken limb buds. (A) Expression patterns of *Hoxa13* and *Hoxd11* and mRNA steady-state levels in chick FL and HL buds from HH20 to HH22. A stronger expression of *Hoxa13* is observed in chick HL bud when compared to FL bud (top). mRNA level of *Hoxd11* increases in FL bud as development proceeds yet seems to decrease in HL bud (bottom). Expression levels are normalized to *Gapdh* and shown as fold change relative to FL bud at HH20–21. Error bars indicate standard deviation of three biological replicates. NS, *p* > 0.05; *p < 0.05; **p < 0.01, Welch two-sample *t* test. (B) Expression of *Hoxa13* and *Hoxd11* in mouse FL and HL buds from E10.5 to E10.75. mRNA levels of both genes in FL and HL buds increase as development proceeds. Expression levels are normalized to *Gapdh* and shown as fold change relative to FL buds at E10.5. Error bars indicate standard deviation of two or four biological replicates. *p < 0.05; NS, *p* > 0.05, Welch two-sample *t* test. For both A and B, individual numerical values of RT-qPCR are given in S1 Table. E, embryonic day; FL, forelimb; HH, Hamburger–Hamilton stage; HL, hindlimb; RT-qPCR, quantitative reverse transcription PCR.

We examined this possibility by performing time course WISH analysis and quantitative reverse transcription PCR (RT-qPCR) experiments using chick and mouse entire fore- and hindlimb buds from HH20 to HH22 and E10.5 to E10.75, respectively (Fig 6). Although these developmental stages are not strictly equivalent between chick and mouse [49], they were selected because the size difference between chick fore- and hindlimb buds is not yet too large between HH20 and HH22 [50]. Also, *Hoxa13* starts to be expressed in mouse forelimb buds at around E10.5 [51]. Whereas the onset of *Hoxa13* expression was detected by WISH in chick forelimb bud at HH22, *Hoxa13* transcripts were already well present in chick hindlimb bud at HH20–21 (Fig 6A). Also, the expression level of this gene in hindlimb buds was markedly stronger than in forelimb buds (*p*-values = 2e-3 for both stages, Fig 6A, right). *Hoxa11* expression was also higher in chick hindlimb buds than in forelimb buds (*p*-values = 7e-3 for both stages, S7A Fig), as was also observed in the RNA-seq dataset, with FPKM values from 27 to 61 (S1 Table), suggesting that the entire chicken *HoxA* cluster was activated in hindlimb buds before it was switched on in forelimb buds. This was nevertheless not a general phenomenon for *Hox* genes, and the expression of *Hoxd13*, for example, was comparable between fore- and hindlimb buds (S7C Fig, S1 Table).

In the mouse, the development of the forelimb bud precedes that of hindlimb buds by about half a day. In contrast, the initiation of both fore- and hindlimb bud in chicken is almost concomitant, and the growth of the hindlimb bud exceeds that of the forelimb bud [34,50]. However, even when considering these developmental differences, the dramatic variations we scored between both the timing of *Hoxa13* activation and its transcript levels between the chick fore- and hindlimb buds were different from the situation observed in murine fore- and hindlimb bud (Fig 6B), and an inverse correlation was observed between the activation of *Hoxa13* on the one hand and the down-regulation of *Hoxd* genes such as *Hoxd11* in chick hindlimb bud on the other hand. This was observed neither in chick forelimb bud nor in mouse limb buds, supporting the idea that an early activation of *Hoxa13* induces a premature termination of T-DOM activity in chick hindlimb bud.

We asked whether the profiles from H3K27ac chromatin immunoprecipitation sequencing (ChIP-seq) and Hi-C data obtained from chick limb tissues covering the *HoxA* cluster would reveal traces of this early and strong activation of *Hoxa13* seen in chick hindlimb buds at HH20 (S5A, S5C and S7D Figs). Whereas this activation was consistent with enriched H3K27ac marks over the *HoxA* cluster itself, it was not fully consistent with the distribution of chromatin marks over those enhancers previously described to regulate *Hoxa13* in developing mouse limbs [16].

### Different impacts of T-DOM upon mouse fore- and hindlimb bud developments

The importance of the T-DOM TAD for mouse proximal limb development was initially assessed in forelimbs exclusively [17]. The fact that birds displayed this striking difference in T-DOM-dependent regulations in fore- and hindlimb buds suggested that the function of T-DOM enhancers may be implemented differently in tetrapod fore- and hindlimbs. We investigated this possibility by looking at the effect of a deletion of T-DOM (the *HoxD*^*Del[attp-SB3]*^ allele) upon *Hoxd* gene regulations in both murine fore- and hindlimb buds. We analyzed *HoxD*^*Del(attp-SB3)*^ mouse limb buds in which an approximatively 1-Mb region including T-DOM, as well as its distal border, was deleted.

**Fig 7:**
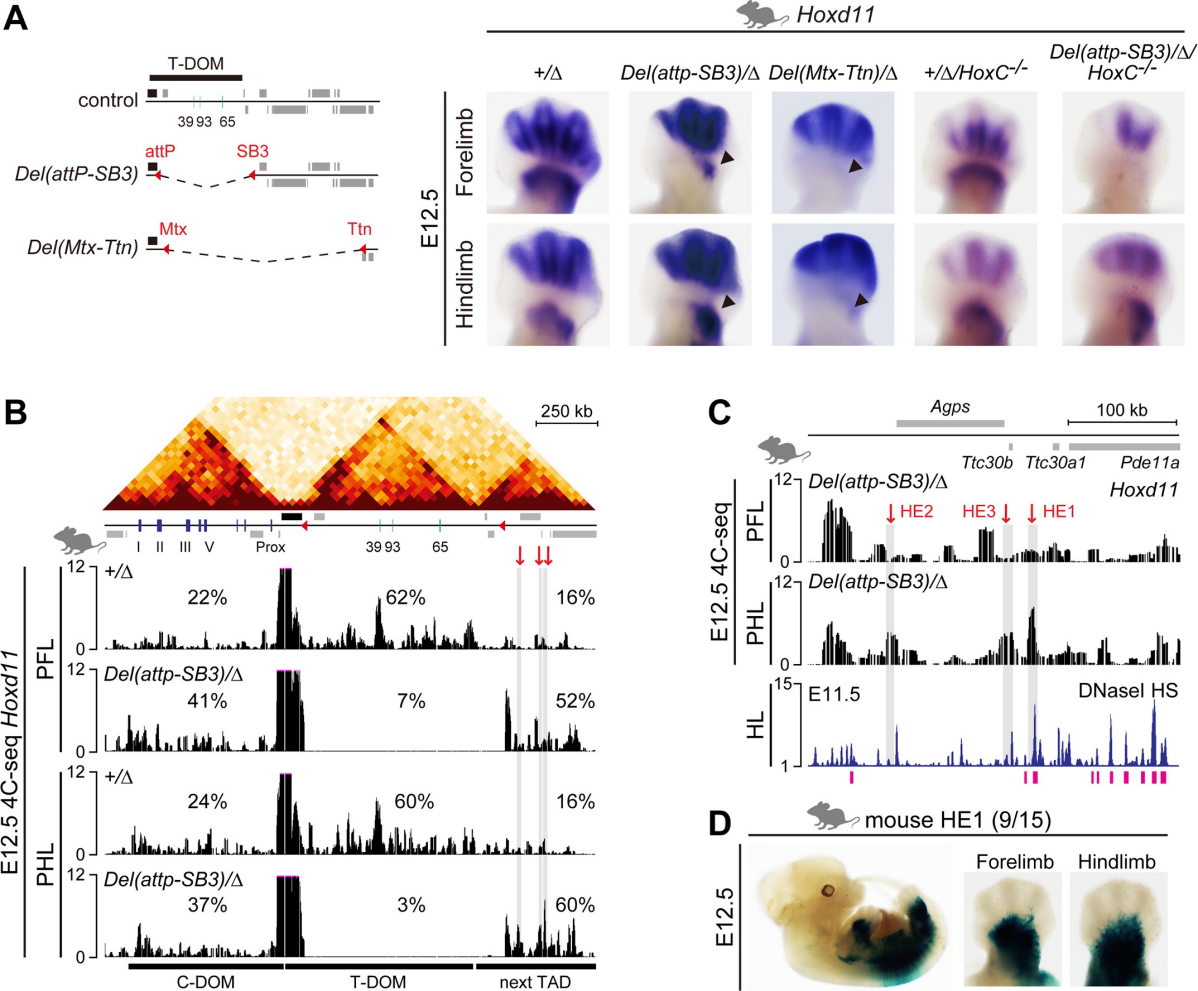
Different effects of a T-DOM deletion on FL and HL buds. (A) The *HoxD*^*Del(attp-SB3)*^ and *HoxD*^*Del(Mtx-Ttn)*^ alleles are deletions of about 1 Mb or 2.1 Mb, respectively, including T-DOM (left, dashed line). *Hoxd11* expression in E12.5 FLs and HLs from either control (*HoxD*^*Del[8–13]/+*^) animals (indicated as “*+/Δ*”) or mutant (*HoxD*^*Del[attp-SB3]/Del[8–13]*^, *HoxD*^*Del[Mtx-Ttn]/Del[8–13]*^, *HoxD*^*Del[8–13]/+*^*;HoxC*^*−/−*^, *HoxD*^*Del[attp-SB3]/Del[8–13]*^*;HoxC*^*−/−*^) littermates (indicated as “*Del(attp-SB3)/Δ*,” “*Del(Mtx-Ttn)/Δ*,” “*+/Δ/HoxC*^*−/−*^,” and “*Del(attp-SB3)/Δ/HoxC*^*−/−*^,” respectively). In *Del(attp-SB3)/Δ* mutants, *Hoxd11* expression is dramatically reduced in proximal FLs (arrowhead) but remains robust in proximal HLs (arrowhead). In *HoxD*^*Del(Mtx-TiE2)*^ mutants, *Hoxd11* expression is abrogated in both proximal FL and HL buds (arrowhead). The absence of both T-DOM and the *HoxC* cluster does not affect *Hoxd11* expression. (B) Hi-C data adapted from [19] showing the two TADs on either side of the *HoxD* cluster and the TAD next to T-DOM. The 4C profiles represent contacts established by *Hoxd11* in proximal FL and HL buds from control or *Del(attp-SB3)/Δ* mutant animals. In mutant cells lacking T-DOM (tracks 2 and 4), additional contacts between *Hoxd11* and the neighboring TAD are scored. The shaded region (red arrows) shows the domains in which increased contacts are detected in mutant proximal HL versus proximal FL buds. (C) Enlargement of 4C profiles shown in (B), DNaseI HS profiles using E11.5 embryos and potential limb enhancer regions (pink rectangles) identified by using the Limb-Enhancer Genie tool. Potential HEs are shown by red arrows. (D) Mouse HE1 is active in the proximal FL and HL buds and in the trunk at E12.5. The number indicates stained embryos over total number of integrations. 4C, circular chromosome conformation capture; 4C-seq, 4C sequencing; E, embryonic day; FL, forelimb; HE, hidden enhancer; Hi-C, high-throughput chromosome conformation capture; HL, hindlimb; HS, hypersensitive sites; PFL, proximal FL; PHL, proximal HL; TAD, topologically associating domain; T-DOM, telomeric regulatory domain.

*Hoxd* transcripts produced in E12.5 proximal limbs by the *HoxD*^*Del(attp-SB3)*^ allele (Fig 7A left, *Del[attp-SB3]/Δ*) were scored by both WISH and RT-qPCR (Fig 7A right, S8A Fig, left). In such mutant proximal forelimb buds, *Hoxd11* to *Hoxd8* transcripts were depleted more than 90% when compared to control proximal forelimbs. However, *Hoxd11* transcripts were not as dramatically affected in proximal mutant hindlimbs, and the amounts of *Hoxd10* to *Hoxd8* transcripts were decreased by only 50% to 60% when compared to control animals (Fig 7A right, S8A Fig left). The reduced level of *Hoxd* gene expression resulting from the mouse T-DOM deletion in the forelimb bud thus mimicked the situation observed in chick proximal hindlimb bud (S8A Fig). This deletion also revealed that significant differences exist in the way T-DOM operates in murine forelimb versus hindlimb buds.

The remaining expression of *Hoxd* genes in T-DOM deletion mutant proximal hindlimb buds completely disappeared when a larger deletion was engineered between the *Mtx* and *Titin (Ttn)* genes (Fig 7A), indicating that the genomic regions between SB3 and *Ttn* (i.e., telomeric to the T-DOM TAD) contribute to the difference in gene expression observed between the mouse fore- and hindlimb buds when T-DOM is removed.

To identify potential differences between forelimb and hindlimb in chromatin reorganization after the deletion of T-DOM, we generated 4C profiles from the mutant allele using the *Hoxd11* promoter as a viewpoint (Fig 7B). In control proximal fore- and hindlimb cells, *Hoxd11* mostly contacted the intact T-DOM (60%–62% of contacts, *HoxD* cluster excluded), with a particularly strong interaction with and around the *CS39* region (Fig 7B, tracks 1 and 3). In proximal cells deleted for T-DOM, interactions within the *HoxD* cluster were increased and ectopic contacts were established (or strongly reinforced) with the newly fused neighboring telomeric TAD (Fig 7B, tracks 2 and 4). As a result, the neighboring telomeric TAD recruited 52% to 60% of contacts (*HoxD* cluster excluded) in proximal cells deleted for T-DOM, as compared to 16% in the control situation. We used this 4C-seq dataset to determine three candidate regions of potential enhancer activity, referred to as hidden enhancer (HE) 1 to 3 (Fig 7B and 7C, red arrows) because of their location outside the T-DOM TAD. We cross-checked this selection with DNaseI hypersensitive sites (HS) data from E11.5 hindlimb buds (GSM1014179) [52], with potential enhancer regions as defined by the Limb-Enhancer Genie tool [53] and with histone H3 lysine 4 monomethylation (H3K4me1) ChIP-seq datasets obtained from control and mutant hindlimb proximal domains (Fig 7C and S8B Fig). Accordingly, HE1 turned out to be the most promising region, and we thus assessed its enhancer potential in transgenic limb buds.

In a transgenic enhancer reporter system, the HE1 region reproducibly drove *lacZ* expression in proximal fore- and hindlimb buds, lateral plate, and somitic mesoderm at E12.5 (Fig 7D and S8D Fig), indicating that the HE1 enhancer activity is not specific for the proximal hindlimb, even though it was potentially active in a hindlimb-specific manner after deletion of T-DOM.

Finally, we looked at potential genetic interactions between the limb-specific differences in *Hoxd* gene expression and the *HoxC* gene cluster. Indeed, *Hoxc11* is strongly transcribed in proximal cells of hindlimb buds (S8C Fig), whereas these transcripts are absent from the equivalent forelimbs territories [54]. Furthermore, in proximal hindlimbs in which *Hoxc* genes are expressed, the amount of *Hoxd* transcripts was decreased by 6- to 26-fold in FPKM when compared to forelimb buds (S1 Table). Also, the deletion of *Hoxc11* on top of *Hoxa11/Hoxd11* double-knockout mice exacerbated the observed hindlimb malformations [11,55], suggesting that HOXC proteins in hindlimb buds may help sustain *Hoxd* transcription. We performed WISH analysis for *Hoxd11* after deleting of the entire *HoxC* cluster [56] on top of the deletion of T-DOM (Fig 7A). In these combined mutant limb buds, expression of *Hoxd11* was still detected in hindlimb proximal cells, indicating that the persistence of *Hoxd11* expression in hindlimb buds in the absence of T-DOM did not depend upon the presence of *Hoxc* transcripts in hindlimb proximal cells.

## Discussion

### Conservation of the bimodal regulation in birds

Although the expression of *Hox* genes belonging to the *HoxA*, *HoxC*, and *HoxD* clusters during limb development are globally comparable between mammals and birds, clear differences are nevertheless apparent. For instance, *Hoxd* gene transcription is reduced in the proximal part of the developing hindlimb buds in birds, i.e., in a cellular domain in which their function is absolutely required for proper mouse hindlimb development [55,57]. Also, although *Hoxd12* is expressed in the mouse limb buds like *Hoxd13* (i.e., mostly under the control of C-DOM), its expression in the proximal avian forelimb buds resembles that of *Hoxd11*, suggesting it is controlled by T-DOM. The impact of these differences in *Hox* gene expression on the variations of limb morphologies is difficult to assess, particularly in the absence of experimental genetics in birds. Unlike in developing spines, in which a clear correspondence was established between *Hox* transcript domains and differences in vertebral formula in birds and mammals [58], such a direct relationship is more difficult to propose in the case of limbs for which many other genetic components are involved on top of *Hox* genes.

Because these expression specificities depend on the implementation of global regulations located within the two TADs flanking the *HoxD* cluster, we wondered whether the structures of these TADs were somehow modified in birds or at least whether they would show some variation either between the two species or between the bird fore- and hindlimb buds. A global analysis of 4C and CHi-C datasets did not reveal any salient differences between mammals and birds regarding the way they implement this complex bimodal limb regulation. The TADs appeared well conserved between the two species, as did the presence in chick of most—if not all—regulatory elements that had been described in the mouse counterparts, on both sides on the gene cluster [17,18], even though the chick TADs were reduced in size. We thus conclude that the bimodal regulatory strategy described in mammals (see [59]) is implemented in a similar manner during bird development, thus reinforcing the idea that the function of *Hox* genes at these early steps of limb development is mostly to set up and organize the basic plan of the future appendages rather than to elaborate or fine-tune a prepatterned structure.

### Interspecies comparison of the TAD boundary at *HoxD*

**Fig 8:**
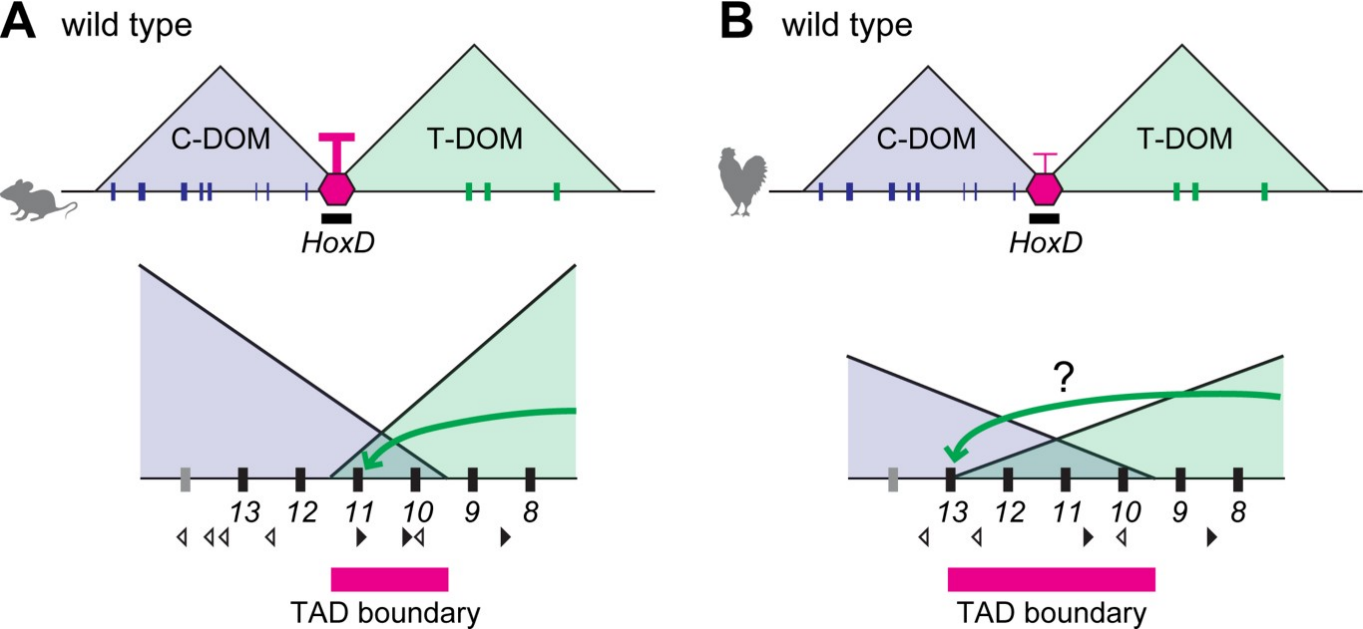
Model of TAD boundaries at the mouse and chicken *HoxD* cluster. (A, B) TAD boundaries at the *HoxD* locus in mouse (A) and chick (B) limb buds. (A) In the mouse, the boundary is dynamic and moves along a few genes within a window determined by a series of CTCF sites. Accordingly, T-DOM enhancers interact with promoters up to *Hoxd11* (green arrow in A). (B) In chick, the boundary appears slightly displaced toward the *Hoxd13* locus. This latter situation may enable T-DOM enhancers to interact with *Hoxd13* more efficiently in chick than in murine limb bud cells (green arrow in B). Black and white arrowheads indicate the orientation of CTCF motifs. C-DOM, centromeric regulatory domain; CTCF, CCCTC-binding factor; TAD, topologically associating domain; T-DOM, telomeric regulatory domain.

Whereas these global controls are thus well conserved amongst tetrapods, the distinct expression of *Hoxd12* in proximal limbs between mouse and chick suggests that the width of the TAD boundary at the *HoxD* locus may vary between the two species. By using transcriptome, 4C, and Hi-C datasets, we previously observed different positions of this boundary in mouse distal versus proximal limb cells because *Hoxd10* and *Hoxd11* respond first to T-DOM and then to C-DOM regulations. We thus proposed that the TAD boundary was located between *Hoxd11* and *Hoxd12* in proximal cells and between *Hoxd9* and *Hoxd10* in distal cells [17,19] (Fig 8A).

In contrast, the chick *Hoxd12* is strongly expressed in proximal forelimb buds, suggesting that the TAD boundary expands toward the 5′ part of the gene cluster, close to *Hoxd13* (Fig 8B). Our CHi-C analysis reinforced this view and positioned this boundary around the *Hoxd13* gene in chick limb buds at early stages (HH20), i.e., when T-DOM is active and controls the first phase of *Hoxd* gene transcription. Subsequently (HH30), the boundary region was localized between *Hoxd13* and *Hoxd12* in chick limbs. Of note, *Hoxd12* is expressed in proximal limbs in geckos as in chicken [23], suggesting that the TAD boundary at the *HoxD* locus in proximal buds may have been shifted during tetrapod evolution between birds and squamates on the one hand and mammals on the other hand.

TAD boundaries at *Hox* loci may thus act as morphological cursors that could redistribute the various subsets of *Hox* genes responding to either proximal or distal enhancers. These differences in boundary position may rely upon distinct distribution and/or usage of CTCF binding sites. In the mouse, subsets of genes responding to either proximal or distal limb enhancers are delimited by different sets of bound CTCF sites [19] (Fig 8). Here, we show that chicken forelimb bud cells have fewer bound CTCF sites in the *HoxD* cluster than their murine counterparts, which could modulate the positioning of the boundary. This decrease in the overall strength of the boundary effect as a result of having fewer sites occupied by CTCF may account for the visible extension of interactions up to *Hoxd12-Hoxd13* established by proximal enhancers (Fig 8). This hypothesis could nevertheless not be verified on chicken hindlimb proximal cells, as these cells do not strongly express the genes controlled by T-DOM.

### Distinct T-DOM regulations in mouse, chick, and bat fore- and hindlimb buds

During bat limb development, *Hoxd10* and *Hoxd11* transcripts are progressively lost throughout the hindlimbs only, in part because of the distinct enhancer activity of the BAR116 sequence located within T-DOM [6]. When the mouse BAR116 cognate sequence was used in a transgenic assay, no activity was detected in any limb cells. Likewise, when we used mouse *CS9* (i.e., a shorter fragment of the *CS93* sequence), staining was not observed. However, when the full-length *CS93* sequence was injected, a robust enhancer activity was scored in a proximal limb region (Fig 3). This discrepancy between two experiments involving almost the same sequences may be caused by the positions of the regions used for the mouse transgenic enhancer assays, the mouse sequence being slightly larger at one of its extremities. Either the enhancer activity was provided by this subfragment or this fragment may be required for the expression of a more widespread activity of the full DNA sequence. It remains that the BAR116 enhancer may not be specific for bats.

However, whereas the bat BAR116 showed strong enhancer activity in forelimb and weak in hindlimb, the mouse equivalent displayed similar enhancer activities between fore- and hindlimbs, in agreement with the continuous expressions of *Hoxd10* and *Hoxd11* in both fore- and hindlimbs. To further validate this correspondence, we looked at the behavior of the chick *CS93* sequence. Although two sets of patterns were obtained with various distal-to-proximal distributions of the *lacZ* staining, a clear imbalance was scored between forelimb and hindlimb cells, with a stronger expression in the former than in the latter. Therefore, the chick enhancer sequence behaved more like the bat sequence than like their murine counterparts. This was supported by the sequence alignments, which revealed more similarities between chick and bats than between the two mammalian species. This similarity correlates with *Hoxd* gene expression and may relate to the large morphological distinctions between fore- and hindlimbs.

### Premature termination of T-DOM regulation in chick hindlimb buds

The termination of the T-DOM enhancer activity in proximal limb cells coincides with the binding of the HOXA13 protein at various sites within the TAD. Also, the removal of both *Hoxa13* and *Hoxd13* functions leads to the continuation of T-DOM regulation and to the failure in C-DOM activation, suggesting that HOX13 proteins are necessary to terminate T-DOM function and to implement the bimodal switch [20,48]. The chick *Hoxd13* gene starts to be expressed at around stage HH18–19 [31], when H3K27ac enrichment is not yet detected over C-DOM (except for *island I*) (Fig 4). Instead, H3K27me3 marks are still present over C-DOM at this early stage, unlike in the early mouse limb buds [17], suggesting that *Hoxd13* early activation in chick may be driven by the T-DOM regulation until the C-DOM regulation is implemented and takes it over. This idea is supported by our CHi-C analysis showing that the TAD boundary is moved toward *Hoxd13* in the early chick limb buds.

In addition, a major difference was observed in the activation of *Hoxa13* between chick and mouse hindlimb buds, with an earlier and stronger activation in chick hindlimb buds at HH20 when compared to both mouse hindlimb buds and chick forelimb buds. This suggests that T-DOM activity may be readily terminated by the premature presence of the HOXA13 protein. Consequently, C-DOM regulation may be implemented earlier in chick hindlimb buds than in forelimb buds. The potential causes for both this early activation of *Hoxa13* in chick hindlimb buds and the strong level of H3K27me3 observed over C-DOM in chick fore- and hindlimb buds remain to be determined.

### Enhancer reallocation and anterior–posterior (AP) position of the limb buds

In mice, the deletion of T-DOM has different effects upon *Hoxd* gene transcription in forelimb and hindlimb proximal cells. Substantial numbers of transcripts indeed persisted only in the proximal hindlimb domain. Since a deletion including more telomeric sequences totally abrogated *Hoxd* expression, we concluded that additional hindlimb-specific enhancers may be located telomeric of T-DOM. The interaction profiles established after the deletion of T-DOM revealed novel hindlimb-specific contacts between *Hoxd* genes and the newly identified HE1 sequence, which is located near the *Agps* and *Pde11a* genes and is thus positioned outside T-DOM but brought to the vicinity of the cluster after the deletion. *Agps* is involved in the rhizomelic chondrodysplasia punctate 3 (RCDP3) condition, with a shortening of proximal limbs [60,61], suggesting that HE1 may be involved in the regulation of *Agps*. The deletion of T-DOM may thus reallocate part of the HE1 proximal limb enhancer activity toward *Hoxd* promoters.

Our genetic approach, however, makes it difficult to assess whether this sequence is used for *Hoxd* regulation under normal circumstances or, alternatively, whether the interactions observed are mostly due to its new proximity to the target genes induced by the deletion of T-DOM. In the former case, this may indicate that as in chick and bats, the global C-DOM regulation may be more active in forelimb than in hindlimb buds, and hence, the HE1 enhancer may not be necessary. In the case of the mouse, this deficit of regulation during proximal hindlimb development could have been compensated for by evolving additional enhancers outside the TAD. The HE1 sequence is bound by several factors, such as Ying Yang 1 (YY1), proposed to mediate enhancer–promoter contacts at distance in embryonic stem cells (ESCs) [62] or paired-like homeodomain 1 (PITX1), a hindlimb-specific factor [63,64].

Finally, the strong remaining expression of *Hoxd* genes observed in T-DOM-deleted mutant proximal hindlimb cells may merely reflect the history of early limb bud cells. In the wild-type condition indeed, the anterior bud emerges from a field of lateral plate mesoderm (LPM) devoid of transcripts for *Hoxd9*, *Hoxd10*, or *Hoxd11*. In contrast, posterior limb buds derive from LPM cells already expressing these genes, because of their more posterior AP position along the trunk mesoderm. In the absence of T-DOM, expression of these genes would not occur in the anterior buds, because of their repressed state and the lack of appropriate enhancers, whereas expression could be inherited and maintained in the posterior buds through a mechanism independent of T-DOM, perhaps involving the HE1 sequence.

## Materials and methods

### Ethics statement

All experiments involving animals were performed in agreement with the Swiss law on animal protection (LPA), under license no. GE 81/14 (to D. D.), after evaluation by the ad hoc comité consultatif de l'expérimentation animale du Canton de Genève.

### Animal experimentation

Chick embryos from a White Leghorn strain were incubated at 37.5°C and staged according to [50].

### In situ hybridization and colorations

WISH was performed as described previously [65]. For *lacZ* staining, embryos were fixed in 1x PBS (pH 7.39–7.41), 2 mM MgCl_2_, 4% PFA/PBS, 0.2% glutaraldehyde, and 5 mM EDTA for 20 min at room temperature and washed 3 times for 20 min in 1x PBS, 2 mM MgCl_2_, 0.2% NP40, and 0.01% sodium deoxycholate. Samples were stained in 5 mM potassium ferrocyanide, 5 mM potassium ferricyanide, and 0.5 mg/ml X-gal at room temperature overnight, followed by washing solution once and refixation with 4% PFA/PBS at 4°C overnight. For Victoria blue staining, dissected chick limbs were fixed in 10% formalin/PBS overnight and rinsed 3 times with 3% HCl in 70% ethanol over the course of a day. Specimens were stained with 1% Victoria blue with 1% HCl in 70% ethanol overnight. Then, they were rinsed with 3% HCl in 70% ethanol several times and dehydrated with 95% ethanol twice. Limbs were cleared in a mixture of 95% ethanol: methyl salicylate, 2:1 and gradually changed to 95% ethanol: methyl salicylate 1:2 and placed in 100% methyl salicylate.

### Microdissection of chick limbs at HH30

Limb tissues at HH30 were microdissected into acropod (distal), mesopod, and zeugopod (proximal) regions. Only distal and proximal regions were used for RNA-seq and 4C-seq.

### RNA-seq and data analysis

Total RNA was extracted from mouse and chick limb bud tissues using the RNeasy Micro Kit (QIAGEN) following the manufacturer instructions. Libraries were prepared with at least 200 ng of total RNA following Illumina TruSeq stranded mRNA sample preparation guide. Sequencings were performed with 100-bp or 75-bp single-end reads. The data were mapped onto either GRCm38 (mm10) or the International Chicken Genome Reference Consortium Gallus_gallus-5.0 (galGal5) using Tophat2 (Version 2.0.9) [66], and unique mapped reads were extracted. The number of uniquely mapped reads was calculated using FLAGSTAT (SAMtools, Version 0.1.18) [67], and this value was used for the subsequent normalization of all coverage data to be the million reads number. In parallel, the FPKM values were obtained using cufflinks (version 2.2.1 options -I 600000 -F 0.05 -j 0.05—compatible-hits-norm–multi-read-correct—library-type fr-firststrand -m 45 -s 20—min-intron-length 40 with ensembl gtf version 89) [68]. All analyses were processed by our Galaxy server (the Bioteam Appliance Galaxy Edition, https://bioteam.net, https://bioteam.net/products/galaxy-appliance) [69].

### RNA extraction and RT-qPCR

Total RNA was extracted using the RNeasy Micro Kit (QIAGEN), following the manufacturer’s instructions. Total RNA (1 μg) was used for cDNA synthesis with SuperScript VILO (Invitrogen). RT-qPCR was performed on a CFX96 real-time system (BIORAD) using the GoTaq qPCR Master Mix (Promega). Each RT-qPCR was carried out with at least two biological replicates, and experimental information is described in S2 Table. Primer sequences for qPCR are listed in S3 Table.

### 4C-seq and data analysis

The chicken bait sequences used for 4C-seq were positioned as close as possible to the equivalent positions in the murine genome. In the case of the “*Hoxd10*/*Hoxd11*” viewpoint, however, a closely corresponding position was made impossible by the distribution of restriction sites, and a bait slightly more telomeric than the mouse bait was thus selected. Each mouse and chick limb tissue was fixed separately with 2% formaldehyde, lysed, and stored at −80°C. Samples were digested with *NlaIII* and *DpnII* as primary and secondary restriction enzymes, respectively, and ligation steps were performed using highly concentrated T4 DNA ligase (Promega) [70]. Inverse PCRs for amplification were carried out using primers for each viewpoint [71] (S3 Table). PCR products were multiplexed and sequenced with 100-bp single-end reads, followed by postprocessing (demultiplexing, mapping, and 4C analysis) using the HTS station (http://htsstation.epfl.ch) [72]. Fragment scores were normalized to the mean score of fragments falling into a region defined as the bait coordinated ± 1 Mb—except with the *HoxD*^*Del(8–13)/+*^ or *HoxD*^*Del(attp-SB3)/Del(8–13)*^ alleles, for which ±2 Mb was used—and the data were smoothed using a running mean with a window size of 11 fragments. The information regarding fragments excluded during the procedure is provided in S3 Table.

Signals falling either into the *HoxD* telomeric or centromeric domains or into the next were assessed by summing the signal in each fragment (before smoothing) overlapping the region of interest and normalized by the sum of signal into both C-DOM and T-DOM domains, except when the *HoxD*^*Del(8–13)/+*^ or *HoxD*^*Del(attp-SB3)/Del(8–13)*^ alleles were used, in which case the signals were normalized by the sum of signals into C-DOM, T-DOM, and the next TAD. Genomic coordinates used for the specific regions are in S3 Table ±10 kb. For domains:

galGal5, chr7: 15,920,642–16,318,067 / chr7: 16,414,183–16,699,172;
mm10, chr2: 73,921,943–74,648,943 / chr2: 74,765,943–75,601,943;
and chr2:75,601,943–76,681,943 for the next TAD.

The differences of contact between specific regions were statistically tested with a Wilcoxon signed rank test using the signal in each fragment using R (http://R-project.org).

### ChIP-seq and data analysis

ChIP experiments were performed as previously described [20]. Microdissected limb tissues from mouse and chick embryos were cross-linked with 1% formaldehyde/PBS for 15 min at room temperature. Chromatin was sheared and used for each immunoprecipitation with anti-H3K27ac (ab4729, Abcam), anti-H3K27me3 (07–449, Merck Millipore), anti-H3K4me1 (ab8895, Abcam), and anti-CTCF (61311, Active Motif). Libraries were prepared with at least 5 ng of purified DNA following the Illumina TruSeq ChIP library preparation guide. Sequencing was performed with 100-bp single-end reads. Demultiplexed ChIP-seq reads were mapped onto the galGal5 or mm10 using Bowtie (Version 0.12.7) [73], with parameters “-m1 –strata–best” according to conditions described previously [74], and PCR duplicates were removed from mapped reads using SAMtools (Version 0.1.18) [67]. By using bamCompare (Version 2.5.0 options—binSize 25—pseudocount 0.5—extendReads 300) [75], the ChIP data from H3K27ac, H3K27me3, and H3K4me1 and the input data were normalized and compared to compute the log2 ratio of the normalized number of reads. In order to quantify the enrichment over regions, the coverage was assessed using multiBamSummary (Version 2.5.0 options—extendReads 300), and the enrichment was calculated as log2 ratio of the normalized number of reads. The CTCF ChIP data were normalized to obtain 1x depth of coverage by using bamCoverage (Version 2.5.0) [75,76]. The CTCF motif orientation analysis was performed as previously described [19]. All analysis was done with our Galaxy server (the Bioteam Appliance Galaxy Edition, https://bioteam.net, https://bioteam.net/products/galaxy-appliance) [69].

### SureSelect probe design and CHi-C

The library of SureSelect enrichment probes was designed over the genomic interval (galGal3: chr7:15,990,001–19,170,000) using the SureDesign online tool of Agilent. Probes cover the entire genomic region (galGal5, chr.7: 14,946,000–17,870,000) and were not designed specifically in proximity of *DpnII* sites. Dissected tissues were dissociated in 10% FCS/PBS with collagenase (C7657, Sigma) to a final concentration of about 1.3 μg/μl, and samples were incubated in Thermomixer at 37°C at 800 rpm for 20 min. After discarding the supernatant, cells were cross-linked with 1% formaldehyde/PBS at room temperature for 10 min, quenched with glycine, and centrifuged to discard the supernatant. Cells were resuspended with PBS containing proteinase inhibitor and then centrifuged again. After removing supernatant, cells were kept at −80°C before use. Hi-C library preparation was performed as described in [77], with the following changes: (1) Resuspended cross-linked cells in ice-cold Lysis buffer were placed on a rotation wheel at 4°C at 30 rpm for 30 min for cell lysis. (2) For chromatin digestion, 400 U of *DpnII* (R0543M, New England Biolabs) was added to the samples and incubated at 37°C at 700 rpm for 4 hr. Another 400 U of *DpnII* was added, and samples were incubated overnight. (3) Blunt-end ligation of biotin filled-in DNA was performed at room temperature at 30 rpm on a rotating wheel for 4 hr. (4) No removal of biotin from unligated ends was performed. (5) DNA was sheared to a size of 200 to 800 bp by using COVARIS E220, with the following conditions; 175W, 10% Duty factor, 200 Cycles per Burst, 60 s. (6) DNA pull-down was performed using Dynabeads MyOne Streptavidin T1 (65601, Thermo Fisher). (7) DNA was measured by Qubit, and 200 ng was used for further treatment, followed by the manufacturer’s protocol (SureSelect^XT^ Target Enrichment System for Illumina Paired-End Multiplexed Sequencing Library).

### CHi-C data analysis

Paired-end sequencing data were processed as follows. First, adapters were removed using cutadapt version 1.6 [78] with the following parameters: -a AGATCGGAAGAGCACACGTCTGAACTCCAGTCAC for R1 and -a AGATCGGAAGAGCGTCGTGTAGGGAAAGAGTGTAGATCTCGGTGGTCGCCGTATCATT for R2. They were then processed by using hicup version 6.1.0 with the bowtie2 version 2.2.6 [79] and SAMtools version 1.2 [67], with galGal5 as reference genome and GATC as restriction enzyme recognition sequence. The pairs were next converted from bam to tabulated files, with the position of the middle of the fragment to which hicup assigned the read, by using an ad hoc python script (available upon request). Only valid pairs with both MAPQ above 30 were kept. Then, pairs with both mates in the capture region (galGal5, chr7:14,946,000–17,870,000) were extracted and processed with cooler to obtain a balance matrix of the capture region with 5-kb bins. The Fig 5 and S7 Fig data were obtained with personal R scripts (available upon request). Fig 5B is the balanced matrices with linear scale. Fig 5D was obtained by subtracting the two balanced matrices. To assess the significance of increased contact between two regions, a Wilcoxon signed rank test was performed using R with the values of the bins in the region of the two balanced matrices. Because 75% of valid pairs MAPQ30 do not involve the capture region, all valid pairs were also processed with cooler to obtain a balance matrix of the whole chromosome 2 at 40 kb. These matrices were used in S7D Fig. To define TAD borders, the TopDom algorithm [42] was run with a window size of 28 from the 10-kb binned balanced matrices, as gaps were too numerous at a 5-kb resolution.

### Mutant stocks

The *HoxD*^*Del(8–13)*^ and *HoxD*^*Del(attp-SB3)*^ alleles were previously described [17,80]. The *HoxD*^*Del(Mtx-Ttn)*^ allele was produced by TAMERE using the *Ttn* exon 2 (TiE2) allele [81] (kindly provided by Dr. Michael Gotthardt) and an *Mtx2* gene trap allele (https://igtc.org/cgi-bin/annotation.py?cellline=CSI574). The sequences of genotyping primers are indicated in S3 Table. All embryos analyzed in Fig 7 and S8 Fig were heterozygotes and balanced by the *HoxD*^*Del(8–13)*^ allele.

### Analysis of sequence alignment and limb enhancer prediction

To characterize the chicken *HoxD* regulatory landscapes, we selected 80 regions from the cognate mouse locus containing potential enhancers in both C-DOM and T-DOM and use LiftOver tool in UCSC. We found 72 regions conserved in the chick genome and located at the same respective positions, whereas 8 regions failed to be identified in the chick, likely because of their partial or full absence (S4 Table). As chicken *island IV* was partially deleted, we divided the mouse *island IV* sequence and used them for LiftOver separately. In this way, we could identify a split *island IV* region in the chicken genome. mVista tools for comparative genomics was used for comparison between sequences of the mouse *CS93* (mm10, chr2: 75,208,103–75,210,328), the bat BAR116 (Myoluc2, GL429772: 6,606,808–6,608,652), and the 2-kb region containing the chick *CS93* (galGal5, chr7: 16,104,863–16,106,863), using the LAGAN alignment program with default parameter (http://genome.lbl.gov/vista/index.shtml). Potential limb enhancer regions were identified by using the Limb-Enhancer Genie tool, with the following condition: (1) analysis type: Scan for top, (2) method: Combined Model (https://leg.lbl.gov/) [53].

### Enhancer transgenic assays

For the enhancer assays, embryos carrying the mouse *CS93/lacZ* and *HE1/lacZ* were generated by lentivirus-mediated transgenesis and pronuclear injection, respectively. The mouse *CS93* (mm10, chr2: 75,208,104–75,210,328) was amplified from C57BL/6 genomic DNA and cloned into the *pRRL-lacZ* vector, as described previously [17]. Lentiviruses were produced and injected into the perivitelline space of mouse zygotes [32]. The mouse *HE1* (mm10, chr2: 75,959,179–75,960,378) and the region containing the chick *CS93* sequence (galGal5, chr7: 16,104,863–16,106,863) were obtained from B6CBAF1/J and White Leghorn genomic DNA, respectively, and cloned into a *βglobin-lacZ* vector. The construct was injected into mouse oocytes. All transgenic embryos were harvested at E12.5 and used for *lacZ* staining.

## Supporting information

S1 Fig (Related to Fig 1) *Hoxa* gene expression in mouse and chick limb buds. (A, B) Comparison of developmental stages between mouse and chick limb buds. (C, D) Whole-mount in situ hybridization analysis of E12.5 mouse and HH28 chick FL and HL buds with expression of *Hoxa* genes. (C) Expression patterns of *Hoxa11* and *Hoxa13* in mouse FL are similar to HL at E12.5. (D) Stronger expression of *Hoxa11* is observed in the chick proximal HL than in the FL at HH28. (E) Expression patterns of *Hox* genes and cartilage pattern stained with Victoria blue at HH30. (F, G) Transcription profiles of *Hoxa* genes in microdissected proximal and distal domains from either E12.5 mouse (F) or HH30 chick (G) FL and HL buds. Right limbs in (C–E) are oriented proximally to the bottom and distally to the top. The *y* axis represents the strand-specific RNA-seq read counts, normalized by the total number of million mapped reads. E, embryonic day; FL, forelimb; HH, Hamburger–Hamilton stage; HL, hindlimb; RNA-seq, RNA sequencing.
(TIF)

S2 Fig (Related to Fig 3) Comparisons between the bat BAR116 and *CS93* sequences from the mouse, bat, and chick genomes. (A) Sequence similarities between chick *CS93*, bat BAR116, and mouse *CS93*. Both sequences bat BAR116 and mouse *CS93* sequences were aligned with BLAT onto the chick genome. The bat BAR116 is more similar to chick *CS93* than to the mouse counterpart. (B) Mouse *CS93* is active in the proximal fore- and hindlimb buds at E12.5 (red arrows). A reduced activity was also observed in the forelimb proximal region. (C) Chick *CS93* showed differential enhancer activity between fore- and hindlimb buds at E12.5. BAR116, Bat Accelerated Region 116; E, embryonic day.
(TIF)

S3 Fig (Related to Fig 2) Regulatory switch between TADs in mouse and chick limb buds. (A–C) The 4C interaction profiles with chick *Hoxd12* (A), mouse *Hoxd13* (B), and chick *Hoxd13* (C) in mouse (E12.5) and chick (HH30) FLs and HLs. (A) In addition to the *CS93* region, contacts between *Hoxd12* and the *CS39* region were also reduced in chick proximal HL cells. In the distal FL and HL bud cells, *Hoxd12* mainly contacted C-DOM, in contrast to the profile observed with the *Hoxd10-11* bait. (B, C) Both mouse *Hoxd13* and chick *Hoxd13* promoters constitutively interacted with C-DOM. The interaction between *Hoxd13* and either *island III* or *Prox* specifically increased in both mouse and chick distal limbs. 4C, circular chromosome conformation capture; C-DOM, centromeric regulatory domain; E, embryonic day; FL, forelimb; HH, Hamburger–Hamilton stage; HL, hindlimb; TAD, topologically associating domain.
(TIF)

S4 Fig (Related to Fig 4) H3K27ac, H3K27me3, and RNA-seq at *HoxD* in chick limbs. (A) H3K27ac marks (tracks 1 to 2 and 5 to 10) and transcription profiles (tracks 3 and 4) at the *HoxD* locus either in whole, proximal, or distal FL and HL buds. H3K27ac covers 5′ *Hoxd* genes in the HL bud at HH19 and HH20. However, the level of *Hoxd* transcripts was reduced at HH20 (see also S3B Fig, track 4). In proximal HL buds at HH28, a significant decrease in H3K27ac enrichment was detected, which corresponded to the reduction in *Hoxd* expression (track 8). (B) H3K27me3 distribution in either whole, proximal, or distal FL and HL buds at HH20 and HH28. Stronger enrichments were observed in both whole HL buds at HH20 and proximal HL buds at HH28, when compared to the corresponding samples from FL buds. The *y* axis represents the strand-specific RNA-seq read counts, normalized by the total number of million mapped reads. Enrichment (*y* axis) of ChIP is shown as the log_2_ ratio of the normalized number of reads between ChIP and input samples. ChIP, chromatin immunoprecipitation; FL, forelimb; H3K27ac, acetylation of histone H3 lysine 27; H3K27me3, trimethylation of H3K27; HH, Hamburger–Hamilton stage; HL, hindlimb; RNA-seq, RNA sequencing.
(TIF)

S5 Fig (Related to Fig 4) H3K27ac and H3K27me3 profiles and RNA-seq at the chick *HoxA* locus. (A, B) Distributions of H3K27ac and H3K27me3 marks over the *HoxA* cluster and its regulatory elements in either whole, proximal, or distal FL and HL buds at HH19, HH20, and HH28. (A) Stronger enrichment of H3K27ac around the 5′ *Hoxa* genes were observed in HL buds at both HH19 and HH20, whereas fewer marks were scored at HH20, in the region covering the *e10* to *e16* enhancers when compared to FL and HL buds at HH19. At HH28, profiles established from proximal or distal region were comparable between FL and HL buds. (B) H3K27me3 marks did not label 3′ *Hoxa* promoters in forelimb buds at HH20 (track 1). Strong enrichments of H3K27me3 over the *HoxA* regulatory elements were not scored, unlike in both C-DOM and T-DOM at the *HoxD* locus (see also Fig 4B). (C) H3K27ac marks (tracks 1 to 2 and 5 to 10) and transcription profiles (tracks 3 and 4) at the *HoxA* locus in either whole, proximal, or distal FL and HL buds. More H3K27ac marks were detected at 5′ *Hoxa* genes in whole HL buds at both HH19 and HH20, corresponding to higher levels of *Hoxa* gene transcripts in HL buds than in FL buds (red arrows in tracks 3 and 4). (D) H3K27me3 profiles in either whole, proximal, or distal FL and HL buds at HH20 and HH28. The *HoxA* regulatory elements at the chick locus were identified by using mouse coordinates and the LiftOver function of the UCSC genome browser. The *y* axis represents the strand-specific RNA-seq read counts, normalized by the total number of million mapped reads. Enrichment (*y* axis) of ChIP is shown as the log_2_ ratio of the normalized number of reads between ChIP and input samples. C-DOM, centromeric regulatory domain; ChIP, chromatin immunoprecipitation; FL, forelimb; H3K27ac, acetylation of histone H3 lysine 27; H3K27me3, trimethylation of H3K27; HH, Hamburger–Hamilton stage; HL, hindlimb; RNA-seq, RNA sequencing; T-DOM, telomeric regulatory domain; UCSC, University of California, Santa Cruz.
(TIF)

S6 Fig (Related to Fig 5) Chromatin conformation at the chick *HoxD* locus in FL and HL buds and conservation of CTCF sites. (A, B) Transcription profiles and CTCF ChIP-seq by using either whole FL or HL buds at HH20. CTCF distributions were relatively similar between FL and HL buds. A noticeable down-regulation of *Hoxd* gene expression was observed in HL buds when compared to FLs. Opened and closed arrowheads indicate the orientation of the CTCF motives. The *y* axis represents the strand-specific RNA-seq read counts, normalized by the total number of million mapped reads. Enrichment (*y* axis) is shown at the normalized 1x sequencing depth of CTCF ChIP. ChIP, chromatin immunoprecipitation; ChIP-seq, ChIP sequencing; CTCF, CCCTC-binding factor; FL, forelimb; HL, hindlimb; RNA-seq, RNA sequencing.
(TIF)

S7 Fig (Related to Fig 6) Expression of *Hoxa* and *Hoxd* genes in chick and mouse limb buds. (A) *Hoxa11* expression was stronger in chick HL buds than in FL buds (left). (B) Expression of *Hoxd12* in both chick FL buds and mouse limb buds displayed a similar trend. (C) Expression of *Hoxd13* in both chick limb buds and mouse FL buds was similar and slightly distinct from mouse HL buds. (D) Hi-C data at the *HoxA* locus with 40-kb resolution using FL and HL buds at HH20. More contacts were scored between the *HoxA* cluster and its regulatory regions in HL buds than that in FL buds (black rectangle). Expression levels were normalized to *Gapdh* and are shown as fold change relative to FL buds at either E10.5 or HH20-21. Error bars indicate standard deviation of either 3 (chick), 2 (E10.5), or 4 (E10.75) biological replicates. **p < 0.01; *p < 0.05; NS, *p* > 0.05, Welch two-sample *t* test. For A, B, and C, individual numerical values of RT-qPCR are given in S1 Table. E, embryonic day; FL, forelimb; HH, Hamburger–Hamilton stage; Hi-C, high-throughput chromosome conformation capture; HL, hindlimb; RT-qPCR, quantitative reverse transcription PCR.
(TIF)

S8 Fig (Related to Fig 7) A T-DOM deletion induces interactions between HE1 and *Hoxd* genes. (A) Relative expression levels for each *Hoxd* gene in mouse and chick proximal FLs and HLs. Expression levels in mouse and chick proximal FL or HL buds were normalized to *mGapdh* and *chGapdh*, respectively, and are shown as fold change relative to mouse control or chick proximal FLs at E12.5 or HH28. Error bars indicate standard deviation of 3 (control), 2 (mutant), or 3 (chick) biological replicates. **p < 0.01; NS, *p* > 0.05, Welch two-sample *t* test. (B) H3K4me1 profiles obtained from proximal FL and HL buds of either control or *Del(attp-SB3)/Δ* mutant embryos at E12.5. The putative HE1 enhancer was covered by H3K4me1 marks and merged with a predicted enhancer region. (C) *Hoxc11* expression from control and *Del(attp-SB3)/Δ* mutant at E12.5. (left) Expression of *Hoxc11* in proximal HL buds partly overlapped with that of *Hoxd11*. The deletion of T-DOM did not affect *Hoxc11* expression. (D) Mouse HE1 is mainly active in the proximal FL and HL buds and in the trunk at E12.5. A weak activity was also observed in the FL proximal region. Enrichment (*y* axis) of ChIP is shown at the log_2_ ratio of the normalized number of reads between ChIP and input samples. For A, individual numerical values of RT-qPCR are given in S1 Table. ChIP, chromatin immunoprecipitation; E, embryonic day; FL, forelimb; H3K4me1, histone H3 lysine 4 monomethylation; HE1, hidden enhancer 1; HH, Hamburger–Hamilton stage; HL, hindlimb; RT-qPCR, quantitative reverse transcription PCR; T-DOM, telomeric regulatory domain.
(TIF)

S1 Table Quantification of RNA-seq, 4C-seq, and ChIP-seq and individual RT-qPCR values. 4C, circular chromosome conformation capture; ChIP-seq, chromatin immunoprecipitation sequencing; RNA-seq, RNA sequencing; RT-qPCR, quantitative reverse transcription PCR.
(XLSX)

S2 Table Information about samples. (XLSX)

S3 Table DNA sequences of primers used for RT-qPCR analyses, genotyping, and 4C-seq. Custom barcodes (4 bp shown by NNNN) were introduced in between the Illumina adapter sequences and the specific viewpoint sequences in order to multiplex and use different samples with the same viewpoint. 4C-seq, circular chromosome conformation capture sequencing; RT-qPCR, quantitative reverse transcription PCR.
(XLSX)

S4 Table LiftOver of the mouse CS regions to the chicken genome. CS, conserved noncoding sequence.
(XLSX)

S5 Table Public datasets used in this research. (XLSX)

## Abbreviations

4C: circular chromosome conformation capture
4C-seq: 4C sequencing
AP: anterior–posterior
BAR116: Bat Accelerated Region 116
C-DOM: centromeric regulatory domain
CHi-C: capture Hi-C
ChIP: chromatin immunoprecipitation
ChIP-seq: ChIP sequencing
Col2a1: *collagen type II alpha 1 chain gene*
CTCF: CCCTC-binding factor
E: embryonic day
ESC: embryonic stem cell
FPKM: Fragments Per Kilobase of exon model per Million mapped fragments
H3K4me1: histone H3 lysine 4 monomethylation
H3K27ac: acetylation of H3K27
H3K27me3: trimethylation of H3K27
HE: hidden enhancer
HH: Hamburger–Hamilton stage
Hi-C: high-throughput chromosome conformation capture
HS: hypersensitive sites
LPM: lateral plate mesoderm
PITX1: paired-like homeodomain 1
RCDP3: rhizomelic chondrodysplasia punctate 3
RNA-seq: RNA sequencing
RT-qPCR: quantitative reverse transcription PCR
TAD: topologically associating domain
T-DOM: telomeric regulatory domain
Ttn: *Titin*
UCSC: University of California, Santa Cruz
WISH: whole-mount in situ hybridization
YY1: Ying Yang 1
