# Supplementary figures and images for: Similarities and differences in the regulation of HoxD genes during chick and mouse limb development
Source: PLoS Biol. 2018 Nov 26;16(11):e3000004. doi: 10.1371/journal.pbio.3000004 (PMC6283595; doi:10.1371/journal.pbio.3000004)

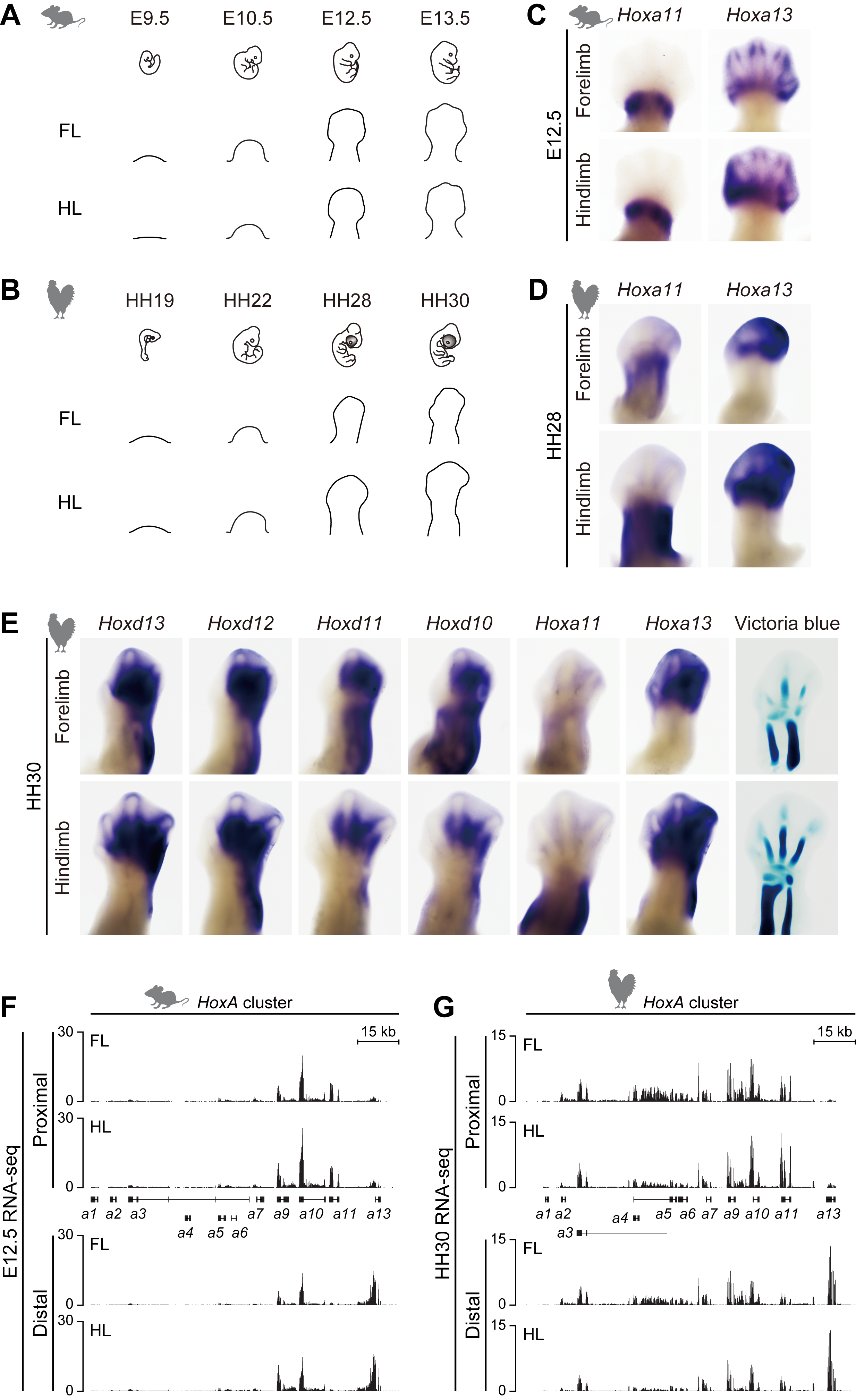

Supplement: S1 Fig — (A, B) Comparison of developmental stages between mouse and chick limb buds. (C, D) Whole-mount in situ hybridization analysis of E12.5 mouse and HH28 chick FL and HL buds with expression of Hoxa genes. (C) Expression patterns of Hoxa11 and Hoxa13 in mouse FL are similar to HL at E12.5. (D) Stronger expression of Hoxa11 is observed in the chick proximal HL than in the FL at HH28. (E) Expression patterns of Hox genes and cartilage pattern stained with Victoria blue at HH30. (F, G) Transcription profiles of Hoxa genes in microdissected proximal and distal domains from either E12.5 mouse (F) or HH30 chick (G) FL and HL buds. Right limbs in (C–E) are oriented proximally to the bottom and distally to the top. The y axis represents the strand-specific RNA-seq read counts, normalized by the total number of million mapped reads. E, embryonic day; FL, forelimb; HH, Hamburger–Hamilton stage; HL, hindlimb; RNA-seq, RNA sequencing. (TIF) [file pbio.3000004.s001.tif]

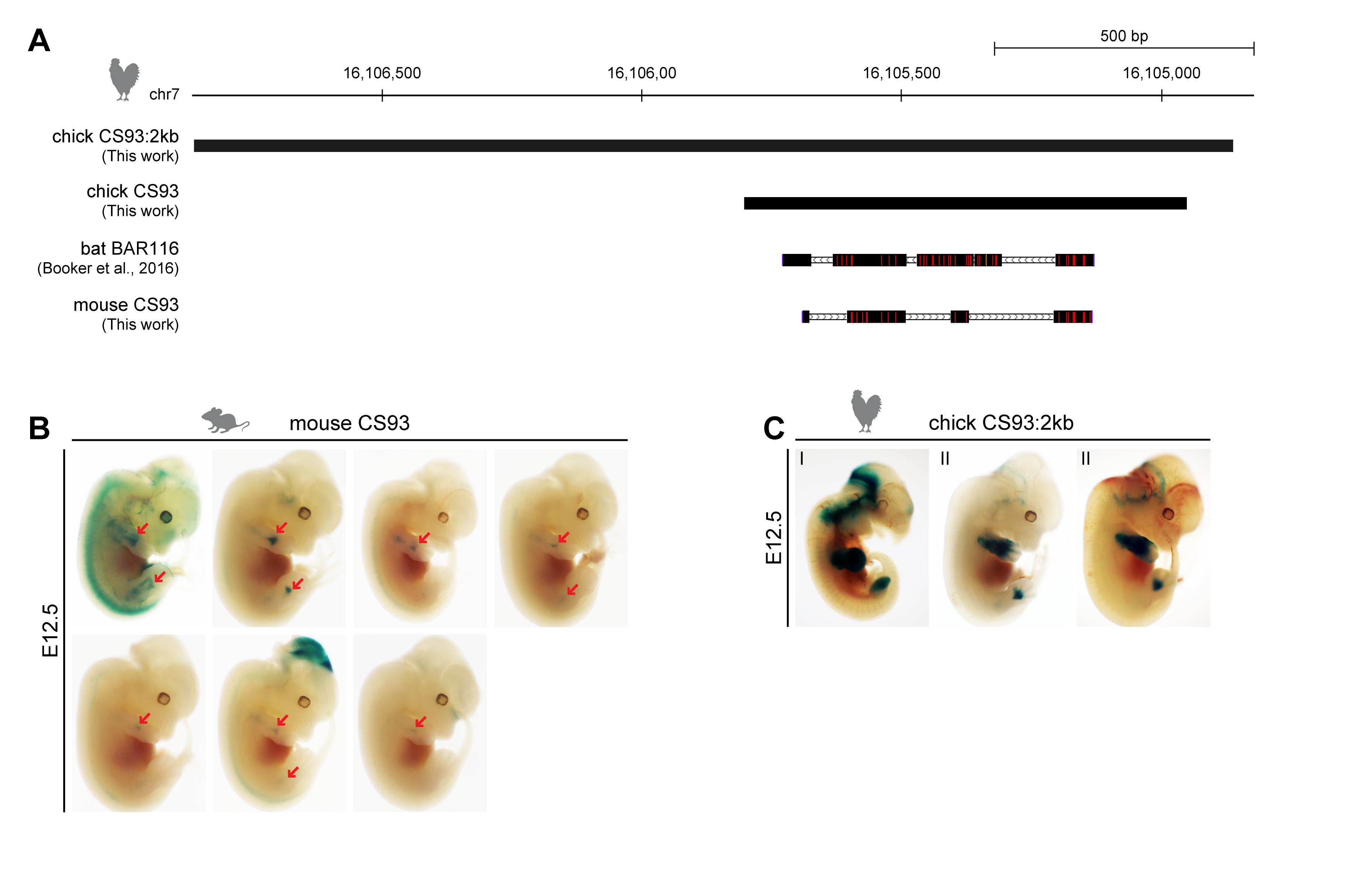

Supplement: S2 Fig — (A) Sequence similarities between chick CS93, bat BAR116, and mouse CS93. Both sequences bat BAR116 and mouse CS93 sequences were aligned with BLAT onto the chick genome. The bat BAR116 is more similar to chick CS93 than to the mouse counterpart. (B) Mouse CS93 is active in the proximal fore- and hindlimb buds at E12.5 (red arrows). A reduced activity was also observed in the forelimb proximal region. (C) Chick CS93 showed differential enhancer activity between fore- and hindlimb buds at E12.5. BAR116, Bat Accelerated Region 116; E, embryonic day. (TIF) [file pbio.3000004.s002.tif]

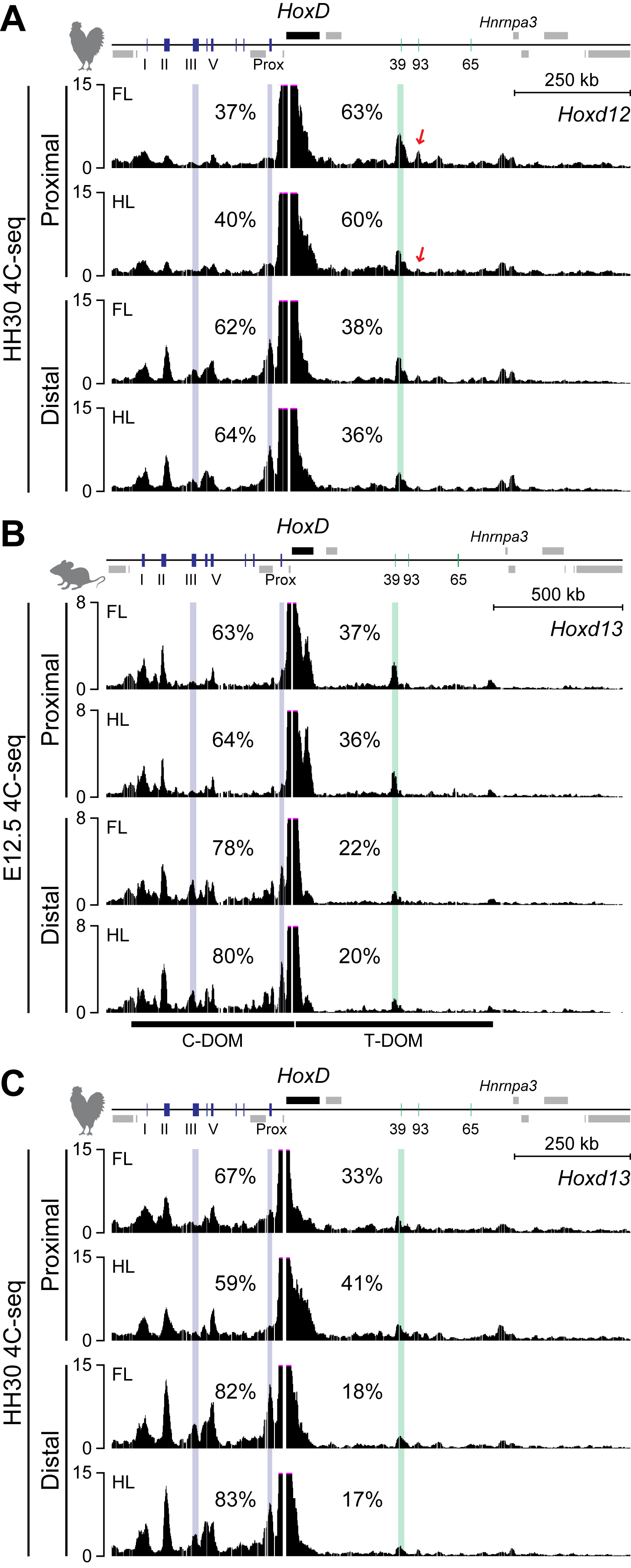

Supplement: S3 Fig — (A–C) The 4C interaction profiles with chick Hoxd12 (A), mouse Hoxd13 (B), and chick Hoxd13 (C) in mouse (E12.5) and chick (HH30) FLs and HLs. (A) In addition to the CS93 region, contacts between Hoxd12 and the CS39 region were also reduced in chick proximal HL cells. In the distal FL and HL bud cells, Hoxd12 mainly contacted C-DOM, in contrast to the profile observed with the Hoxd10-11 bait. (B, C) Both mouse Hoxd13 and chick Hoxd13 promoters constitutively interacted with C-DOM. The interaction between Hoxd13 and either island III or Prox specifically increased in both mouse and chick distal limbs. 4C, circular chromosome conformation capture; C-DOM, centromeric regulatory domain; E, embryonic day; FL, forelimb; HH, Hamburger–Hamilton stage; HL, hindlimb; TAD, topologically associating domain. (TIF) [file pbio.3000004.s003.tif]

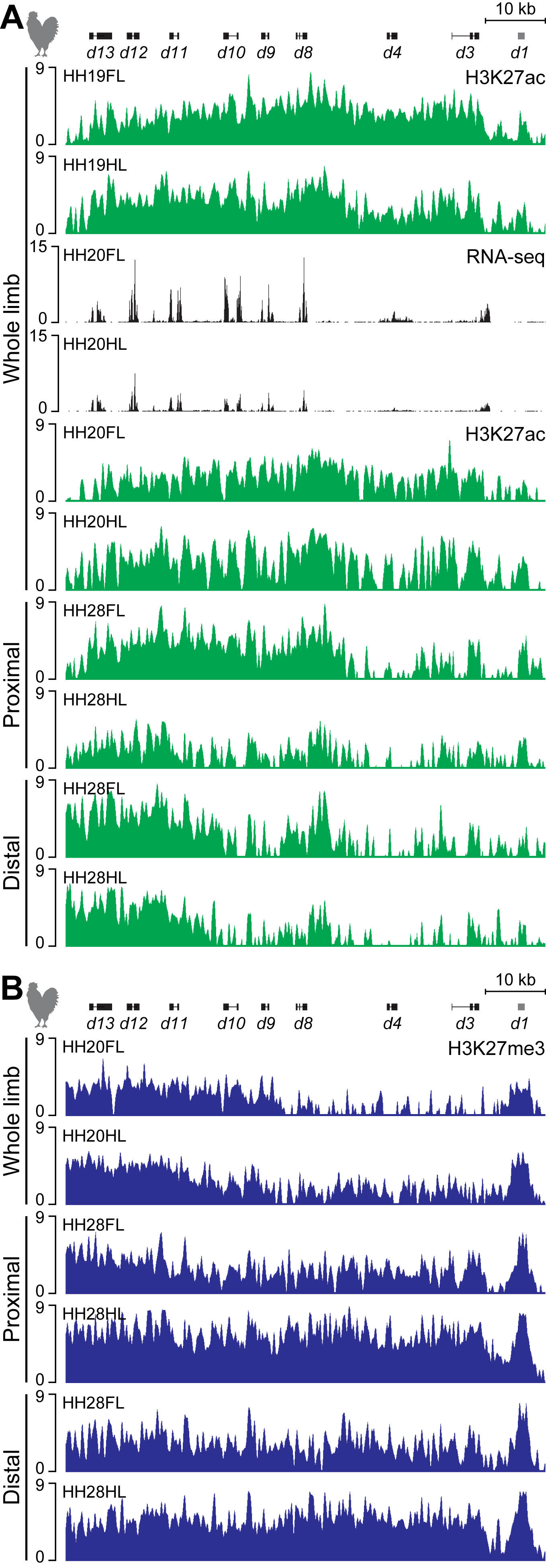

Supplement: S4 Fig — (A) H3K27ac marks (tracks 1 to 2 and 5 to 10) and transcription profiles (tracks 3 and 4) at the HoxD locus either in whole, proximal, or distal FL and HL buds. H3K27ac covers 5′ Hoxd genes in the HL bud at HH19 and HH20. However, the level of Hoxd transcripts was reduced at HH20 (see also S3B Fig, track 4). In proximal HL buds at HH28, a significant decrease in H3K27ac enrichment was detected, which corresponded to the reduction in Hoxd expression (track 8). (B) H3K27me3 distribution in either whole, proximal, or distal FL and HL buds at HH20 and HH28. Stronger enrichments were observed in both whole HL buds at HH20 and proximal HL buds at HH28, when compared to the corresponding samples from FL buds. The y axis represents the strand-specific RNA-seq read counts, normalized by the total number of million mapped reads. Enrichment (y axis) of ChIP is shown as the log2 ratio of the normalized number of reads between ChIP and input samples. ChIP, chromatin immunoprecipitation; FL, forelimb; H3K27ac, acetylation of histone H3 lysine 27; H3K27me3, trimethylation of H3K27; HH, Hamburger–Hamilton stage; HL, hindlimb; RNA-seq, RNA sequencing. (TIF) [file pbio.3000004.s004.tif]

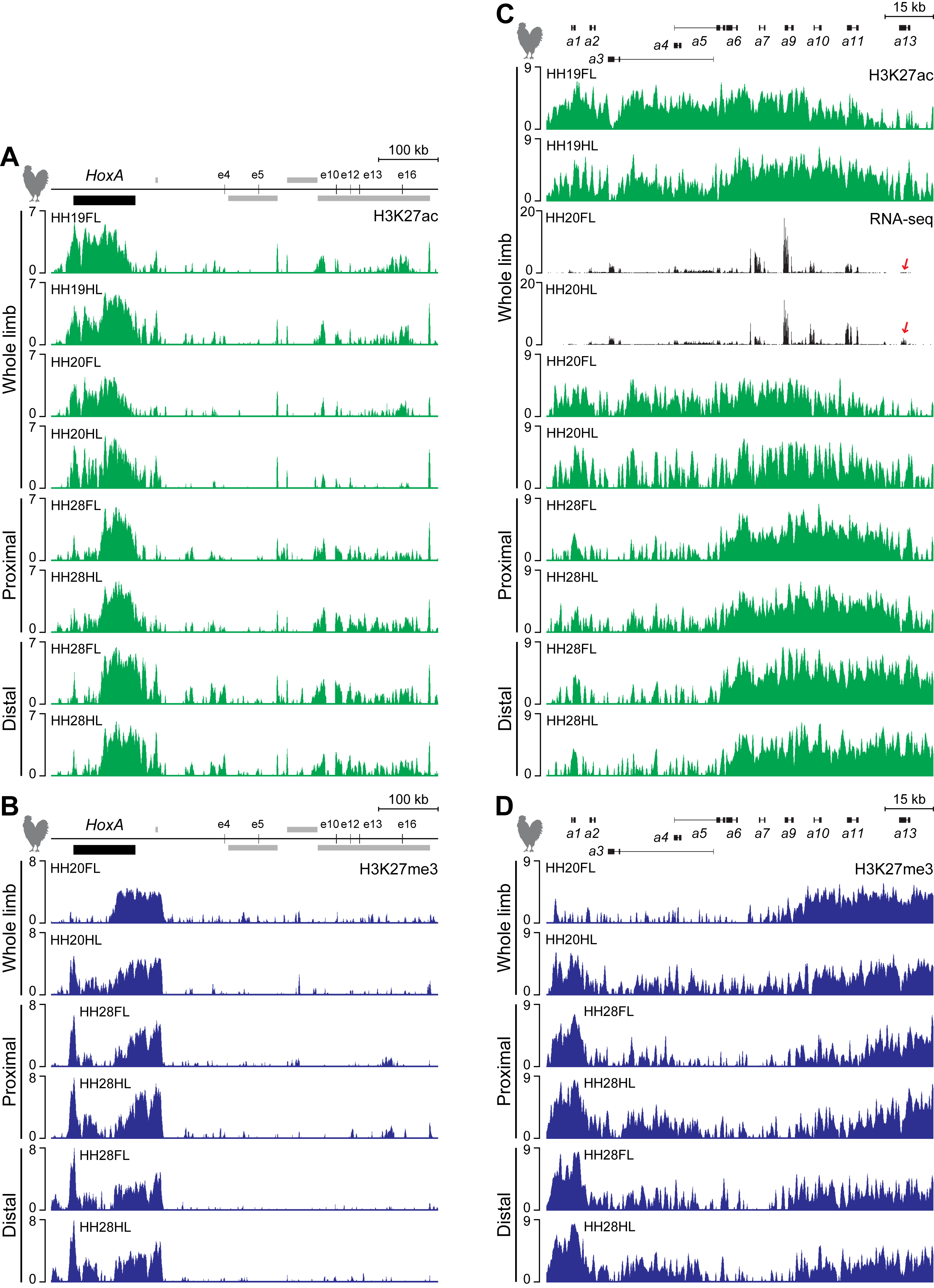

Supplement: S5 Fig — (A, B) Distributions of H3K27ac and H3K27me3 marks over the HoxA cluster and its regulatory elements in either whole, proximal, or distal FL and HL buds at HH19, HH20, and HH28. (A) Stronger enrichment of H3K27ac around the 5′ Hoxa genes were observed in HL buds at both HH19 and HH20, whereas fewer marks were scored at HH20, in the region covering the e10 to e16 enhancers when compared to FL and HL buds at HH19. At HH28, profiles established from proximal or distal region were comparable between FL and HL buds. (B) H3K27me3 marks did not label 3′ Hoxa promoters in forelimb buds at HH20 (track 1). Strong enrichments of H3K27me3 over the HoxA regulatory elements were not scored, unlike in both C-DOM and T-DOM at the HoxD locus (see also Fig 4B). (C) H3K27ac marks (tracks 1 to 2 and 5 to 10) and transcription profiles (tracks 3 and 4) at the HoxA locus in either whole, proximal, or distal FL and HL buds. More H3K27ac marks were detected at 5′ Hoxa genes in whole HL buds at both HH19 and HH20, corresponding to higher levels of Hoxa gene transcripts in HL buds than in FL buds (red arrows in tracks 3 and 4). (D) H3K27me3 profiles in either whole, proximal, or distal FL and HL buds at HH20 and HH28. The HoxA regulatory elements at the chick locus were identified by using mouse coordinates and the LiftOver function of the UCSC genome browser. The y axis represents the strand-specific RNA-seq read counts, normalized by the total number of million mapped reads. Enrichment (y axis) of ChIP is shown as the log2 ratio of the normalized number of reads between ChIP and input samples. C-DOM, centromeric regulatory domain; ChIP, chromatin immunoprecipitation; FL, forelimb; H3K27ac, acetylation of histone H3 lysine 27; H3K27me3, trimethylation of H3K27; HH, Hamburger–Hamilton stage; HL, hindlimb; RNA-seq, RNA sequencing; T-DOM, telomeric regulatory domain; UCSC, University of California, Santa Cruz. (TIF) [file pbio.3000004.s005.tif]

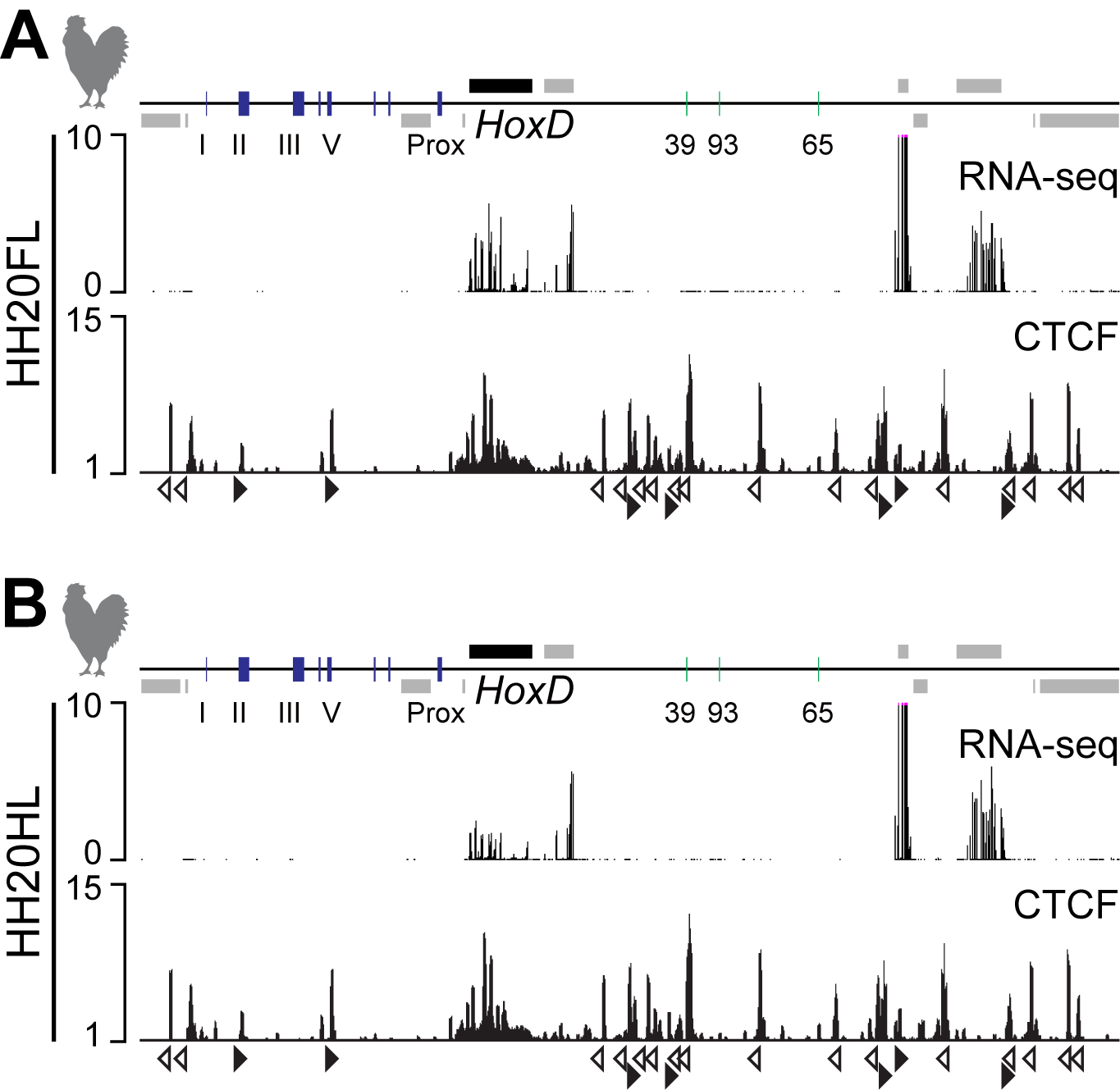

Supplement: S6 Fig — (A, B) Transcription profiles and CTCF ChIP-seq by using either whole FL or HL buds at HH20. CTCF distributions were relatively similar between FL and HL buds. A noticeable down-regulation of Hoxd gene expression was observed in HL buds when compared to FLs. Opened and closed arrowheads indicate the orientation of the CTCF motives. The y axis represents the strand-specific RNA-seq read counts, normalized by the total number of million mapped reads. Enrichment (y axis) is shown at the normalized 1x sequencing depth of CTCF ChIP. ChIP, chromatin immunoprecipitation; ChIP-seq, ChIP sequencing; CTCF, CCCTC-binding factor; FL, forelimb; HL, hindlimb; RNA-seq, RNA sequencing. (TIF) [file pbio.3000004.s006.tif]

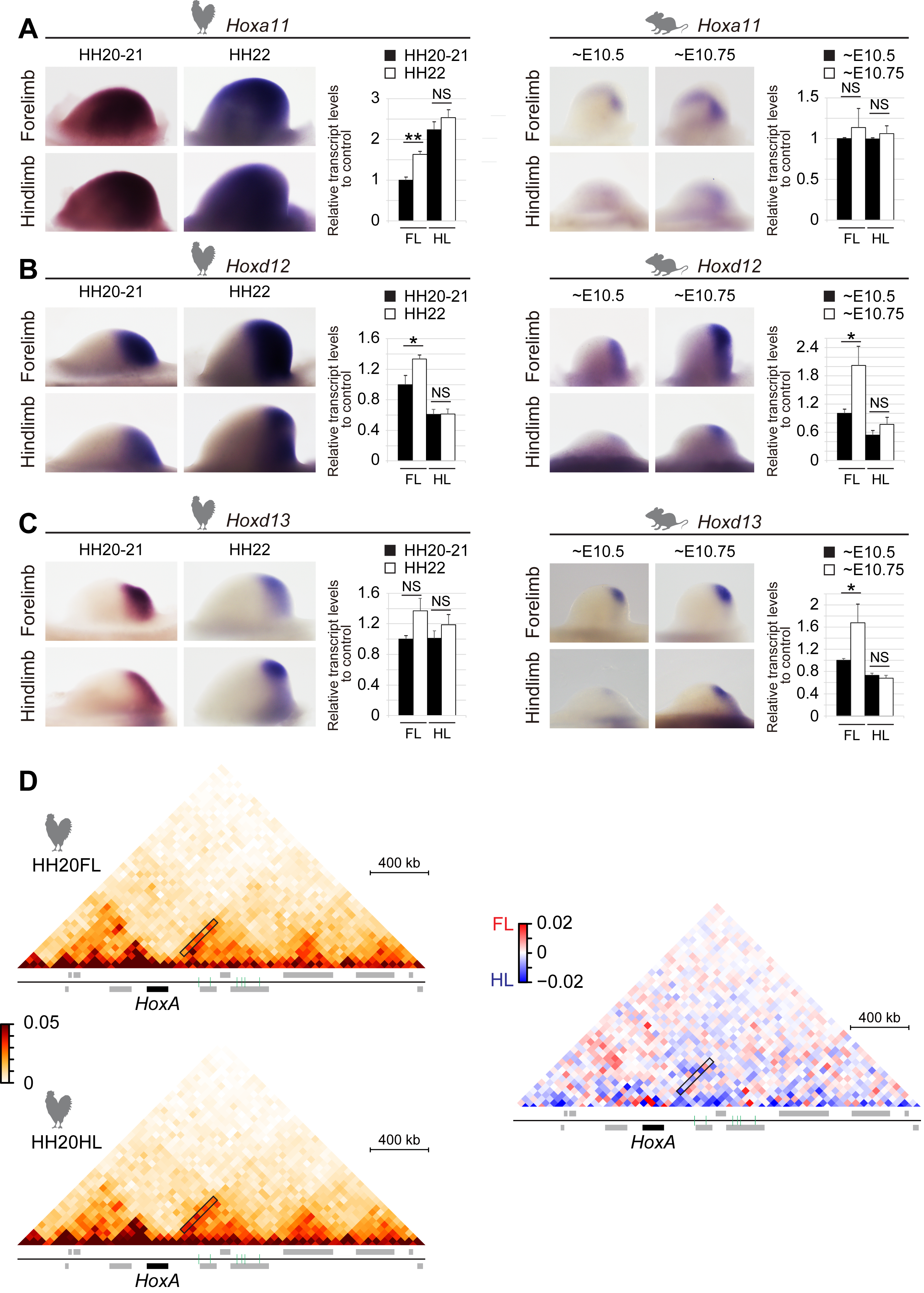

Supplement: S7 Fig — (A) Hoxa11 expression was stronger in chick HL buds than in FL buds (left). (B) Expression of Hoxd12 in both chick FL buds and mouse limb buds displayed a similar trend. (C) Expression of Hoxd13 in both chick limb buds and mouse FL buds was similar and slightly distinct from mouse HL buds. (D) Hi-C data at the HoxA locus with 40-kb resolution using FL and HL buds at HH20. More contacts were scored between the HoxA cluster and its regulatory regions in HL buds than that in FL buds (black rectangle). Expression levels were normalized to Gapdh and are shown as fold change relative to FL buds at either E10.5 or HH20-21. Error bars indicate standard deviation of either 3 (chick), 2 (E10.5), or 4 (E10.75) biological replicates. **p < 0.01; *p < 0.05; NS, p > 0.05, Welch two-sample t test. For A, B, and C, individual numerical values of RT-qPCR are given in S1 Table. E, embryonic day; FL, forelimb; HH, Hamburger–Hamilton stage; Hi-C, high-throughput chromosome conformation capture; HL, hindlimb; RT-qPCR, quantitative reverse transcription PCR. (TIF) [file pbio.3000004.s007.tif]

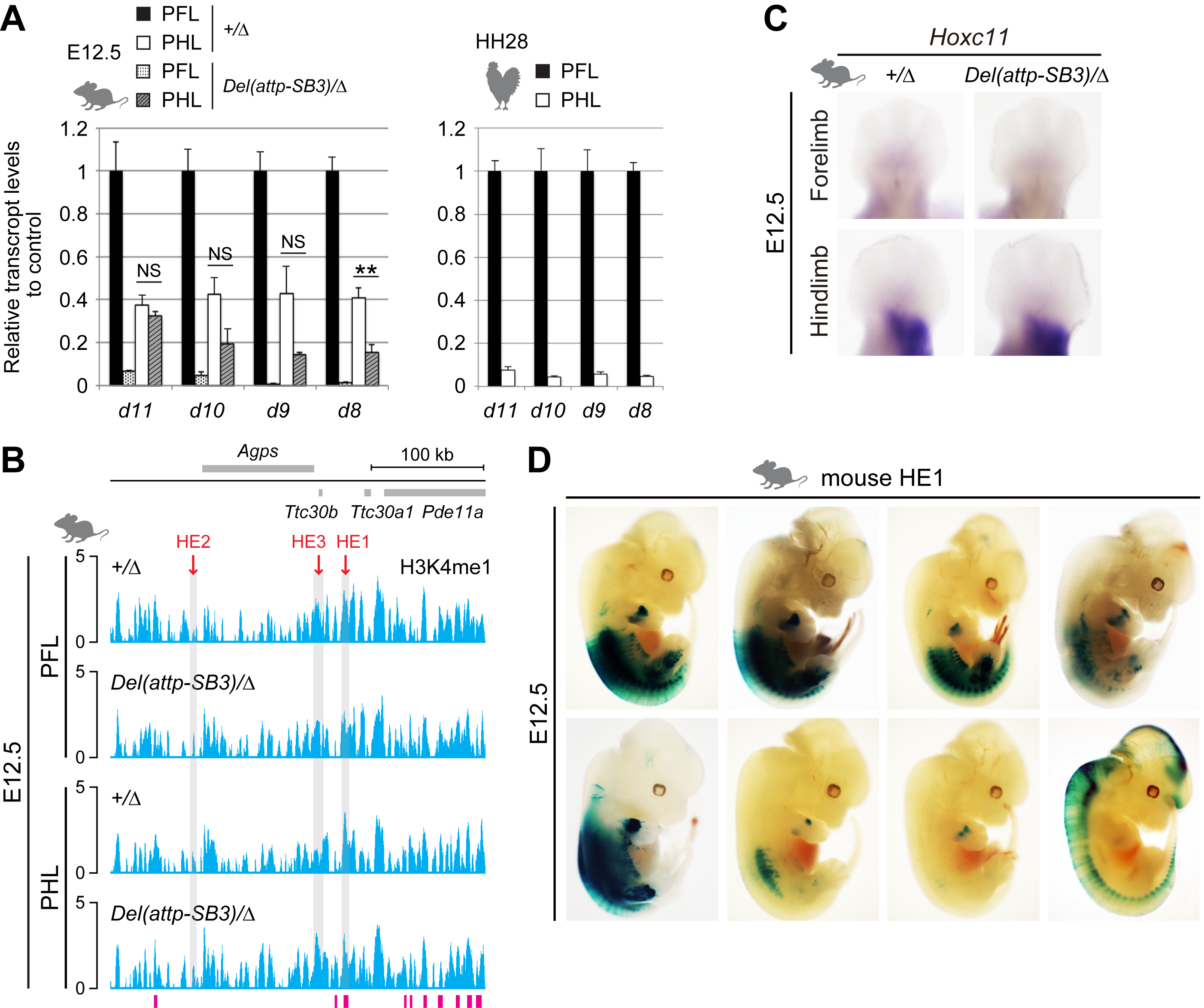

Supplement: S8 Fig — (A) Relative expression levels for each Hoxd gene in mouse and chick proximal FLs and HLs. Expression levels in mouse and chick proximal FL or HL buds were normalized to mGapdh and chGapdh, respectively, and are shown as fold change relative to mouse control or chick proximal FLs at E12.5 or HH28. Error bars indicate standard deviation of 3 (control), 2 (mutant), or 3 (chick) biological replicates. **p < 0.01; NS, p > 0.05, Welch two-sample t test. (B) H3K4me1 profiles obtained from proximal FL and HL buds of either control or Del(attp-SB3)/Δ mutant embryos at E12.5. The putative HE1 enhancer was covered by H3K4me1 marks and merged with a predicted enhancer region. (C) Hoxc11 expression from control and Del(attp-SB3)/Δ mutant at E12.5. (left) Expression of Hoxc11 in proximal HL buds partly overlapped with that of Hoxd11. The deletion of T-DOM did not affect Hoxc11 expression. (D) Mouse HE1 is mainly active in the proximal FL and HL buds and in the trunk at E12.5. A weak activity was also observed in the FL proximal region. Enrichment (y axis) of ChIP is shown at the log2 ratio of the normalized number of reads between ChIP and input samples. For A, individual numerical values of RT-qPCR are given in S1 Table. ChIP, chromatin immunoprecipitation; E, embryonic day; FL, forelimb; H3K4me1, histone H3 lysine 4 monomethylation; HE1, hidden enhancer 1; HH, Hamburger–Hamilton stage; HL, hindlimb; RT-qPCR, quantitative reverse transcription PCR; T-DOM, telomeric regulatory domain. (TIF) [file pbio.3000004.s008.tif]
